# A Multiphase Multiobjective Dynamic Genome-Scale Model Shows Different Redox Balancing among Yeast Species of the *Saccharomyces* Genus in Fermentation

**DOI:** 10.1128/mSystems.00260-21

**Published:** 2021-08-03

**Authors:** David Henriques, Romain Minebois, Sebastián N. Mendoza, Laura G. Macías, Roberto Pérez-Torrado, Eladio Barrio, Bas Teusink, Amparo Querol, Eva Balsa-Canto

**Affiliations:** a (Bio)process Engineering Group, IIM-CSIC, Vigo, Spain; b Systems Biology in Yeast of Biotechnological Interest, IATA-CSIC, Paterna, Spain; c Systems Bioinformatics, Vrije Universiteit Amsterdam, Amsterdam, The Netherlands; d Department of Genetics, University of Valencia, Burjassot, Spain; MIT

**Keywords:** batch fermentation, *Saccharomyces* species, cryotolerant species, dynamic genome-scale models, redox balance

## Abstract

Yeasts constitute over 1,500 species with great potential for biotechnology. Still, the yeast Saccharomyces cerevisiae dominates industrial applications, and many alternative physiological capabilities of lesser-known yeasts are not being fully exploited. While comparative genomics receives substantial attention, little is known about yeasts’ metabolic specificity in batch cultures. Here, we propose a multiphase multiobjective dynamic genome-scale model of yeast batch cultures that describes the uptake of carbon and nitrogen sources and the production of primary and secondary metabolites. The model integrates a specific metabolic reconstruction, based on the consensus Yeast8, and a kinetic model describing the time-varying culture environment. In addition, we proposed a multiphase multiobjective flux balance analysis to compute the dynamics of intracellular fluxes. We then compared the metabolism of S. cerevisiae and Saccharomyces uvarum strains in a rich medium fermentation. The model successfully explained the experimental data and brought novel insights into how cryotolerant strains achieve redox balance. The proposed model (along with the corresponding code) provides a comprehensive picture of the main steps occurring inside the cell during batch cultures and offers a systematic approach to prospect or metabolically engineering novel yeast cell factories.

**IMPORTANCE** Nonconventional yeast species hold the promise to provide novel metabolic routes to produce industrially relevant compounds and tolerate specific stressors, such as cold temperatures. This work validated the first multiphase multiobjective genome-scale dynamic model to describe carbon and nitrogen metabolism throughout batch fermentation. To test and illustrate its performance, we considered the comparative metabolism of three yeast strains of the *Saccharomyces* genus in rich medium fermentation. The study revealed that cryotolerant *Saccharomyces* species might use the γ-aminobutyric acid (GABA) shunt and the production of reducing equivalents as alternative routes to achieve redox balance, a novel biological insight worth being explored further. The proposed model (along with the provided code) can be applied to a wide range of batch processes started with different yeast species and media, offering a systematic and rational approach to prospect nonconventional yeast species metabolism and engineering novel cell factories.

## INTRODUCTION

Yeasts have been used to produce fermented foods and beverages for millennia and are among the most frequently used microorganisms in biotechnology. Saccharomyces cerevisiae dominates the scene and many research efforts focus on engineering this species for particular applications (for examples, see references 1 to 4). Nowadays it is used to produce glycerol (5), biopharmaceutical proteins (6), or secondary metabolites, such as aromas or bioflavors (7, 8).

However, yeasts constitute a large group of 1,500 (so far) described species, and much less attention has been paid to nonconventional yeasts. These species remain a mostly untapped resource of alternative metabolic routes for substrate use and product formation as well as tolerances to specific stressors (9, 10). To exploit these alternatives efficiently, it is essential to understand the metabolic pathways of these species. Given the complexity of the endeavor, a modeling approach becomes indispensable.

Genome-scale models (GEMs) can contextualize high-throughput data and predict genotype-environment-phenotype relationships (11, 12). While GEMs have been widely used for the study and metabolic engineering of S. cerevisiae strains in continuous (steady-state) fermentations (13), their use to predict batch (dynamic) fermentation is still scarce. Nevertheless, many yeast-based processes operate in batch mode.

In batch operation, cell culture follows a growth curve with the following phases: lag phase, exponential growth, growth under nutrient limitation, stationary phase, and cellular decay. Available dynamic GEMs of yeast metabolism focus on the exponential phase and explain reasonably well the measured dynamics of biomass growth, carbon source uptake, and the production of relevant primary metabolites (14–17). The development of GEMs that describe the five phases of batch processes, considering carbon and nitrogen metabolism throughout time and explaining secondary metabolism, is still required.

In this work, we derived a multiphase and multiobjective dynamic genome-scale model of batch fermentation, which accounts for carbon and nitrogen metabolism throughout time and explains secondary metabolism. The model required various refinements to succeed: (i) a novel metabolic reconstruction, based on an extension of the current consensus genome-scale model of S. cerevisiae (Yeast8 [18]); (ii) multiphase multiobjective implementation of a parsimonious flux balance analysis (pFBA) (19) to compute the dynamics of the intracellular fluxes; (iii) a model of protein turnover to explain nitrogen homeostasis; and (iv) a dynamic biomass equation to account for biomass composition variations throughout the process.

As a relevant case study, we considered the metabolism of S. cerevisiae and Saccharomyces uvarum strains in rich medium fermentation. Recent studies revealed that S. uvarum strains show interesting physiological properties. S. uvarum is more cryotolerant than S. cerevisiae, produces more glycerol and less ethanol than S. cerevisiae wine strains, and has different aroma profiles (20–23). In addition, traits such as its increased 2-phenylethanol (24, 25) yield could make this species a good candidate for metabolic engineering studies (26).

We applied the proposed model to investigate the origin of the phenotypic divergence between species. The model explained the experimental data successfully and revealed differences into how species achieve redox balance. Predicted intracellular fluxes led us to hypothesize that cryotolerant yeast strains can use the γ-aminobutyric acid (GABA) shunt as an alternative NADPH source and store reductive power—necessary to subdue oxidative stress under cold conditions—in lipids or other polymers. Additionally, our results are compatible with recent experimental observations showing that most carbon skeletons used to form higher alcohols (i.e., isoamyl alcohol, isobutanol, and 2-phenylethanol) are synthesized *de novo*.

## RESULTS

### Novel metabolic reconstruction.

We updated the Yeast8 consensus genome-scale reconstruction of S. cerevisiae S288C (v.8.3.1) (18) to include 38 metabolites and 50 reactions to explain secondary metabolism (Table S1). Furthermore, comprehensive metabolic annotations, such as SBO (Systems Biology Ontology) terms and MetaNetX identifiers, were added to the new metabolites and reactions.

10.1128/mSystems.00260-21.4 TABLE S1 Novel metabolic reconstruction additions. Table sheet 1 presents the list of reactions and metabolites added to the novel metabolic reconstruction. Among the metabolites added, 13 aroma compounds were included, namely, methionol, ethyl-hexanoate, ethyl-octanoate, 1-hexanol, 1-octanol, 4-tyrosol, hexanal, hexyl-acetate, octyl-acetate, benzyl-acetate, 4-hydroxyphenylacetaldehyde, and 3-methylsulfanylpropanal. Table sheet 2 presents the chemical family (alcohol, ester, or aldehyde) and several aroma descriptors for each of the added compounds. Download Table S1, XLSX file, 0.01 MB. Copyright © 2021 Henriques et al. 2021 Henriques et al. https://creativecommons.org/licenses/by/4.0/ This content is distributed under the terms of the Creative Commons Attribution 4.0 International license.

Among the metabolites added, 13 aroma compounds were included. Noticeably, we found that prior genome-scale reconstructions lacked methionol and tyrosol impeding simulated growth on methionine and tyrosine as sole nitrogen sources, which is known to be possible for several S. cerevisiae strains, including S288C.

MetaDraft, AuReMe, and the results from the orthology analysis were used to create strain-specific models for two wine strains, S. cerevisiae T73 and S. uvarum BMV58, and a strain, S. uvarum CECT12600, found in nonfermentation environments (further details can be found in Text S1). These strains are referred to as ScT73, SuBMV58, and SuCECT12600 here. The three models had 2, 3, and 2 reactions that were not in Yeast8, respectively.

10.1128/mSystems.00260-21.1 TEXT S1 Orthology analysis and genome-scale metabolic reconstruction. The text further elaborates on the steps followed to obtain individual strain reconstructions. Download Text S1, PDF file, 0.3 MB. Copyright © 2021 Henriques et al. 2021 Henriques et al. https://creativecommons.org/licenses/by/4.0/ This content is distributed under the terms of the Creative Commons Attribution 4.0 International license.

### Multiphase multiobjective flux balance analysis framework.

**FIG 1:**
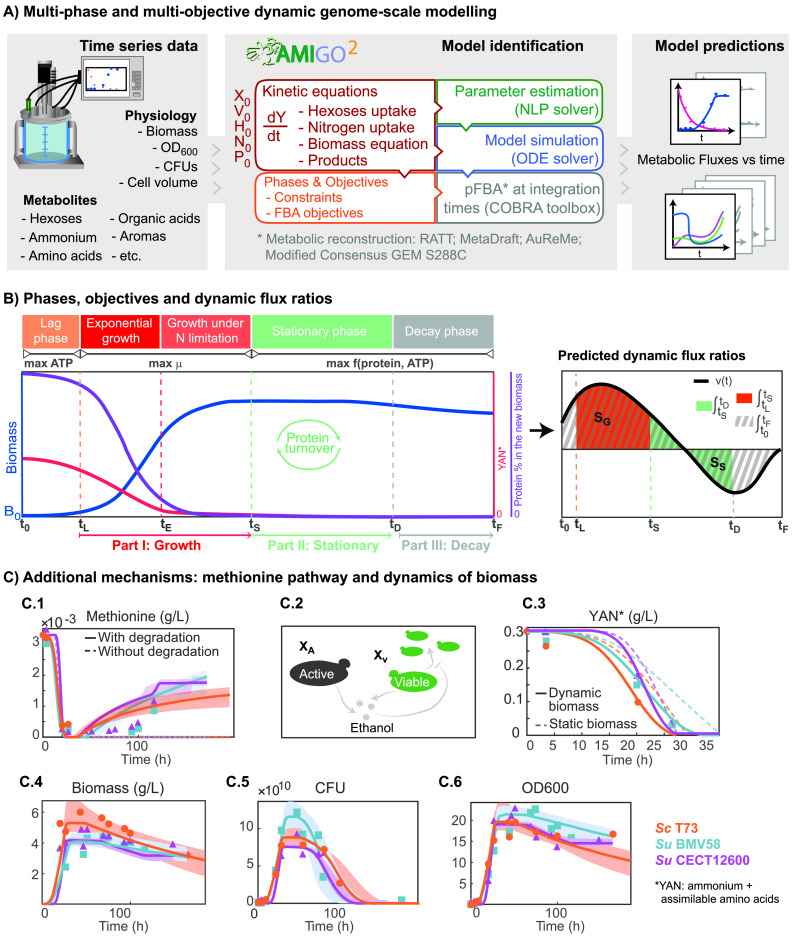
Details on the implementation of the multiphase and multiobjective dynamic genome-scale model to simulate batch fermentation. (A) Implementation, model formulation, and solution approach. (B) Multiphase and multiobjective dynamic FBA and methodology to compute dynamic flux rates. The process starts at *t_0_* = 0 and ends at *t_F_*; the timing of each phase *t_L_*, *t_E_*, *t_S_*, and *t_D_* is computed through parameter estimation. (C) Model improvements through additional mechanisms. (C1) Model prediction versus the experimental dynamics of methionine with and without its degradation pathway; (C2) schematic view of active versus viable biomass in the model; (C3) YAN consumption prediction with static and dynamic biomass equations; (C4 to C6) model predictions versus biomass, CFU, and OD_600_ measurements.

Our results showed that batch fermentation modeling should be divided into five phases in which cellular objectives and flux constraints need to be modified: lag phase, exponential growth, growth under nitrogen limitation, stationary, and decay (Fig. 1A and B sketch the modeling approach). Their duration is imposed by the estimated parameters *t_L_*, *t_E_*, *t_S_*, and *t_D_*, illustrated in Fig. 1B.

Once inoculated, cells encounter new nutrients and undergo a temporary period of nonreplication, the lag phase, during which we assumed that ATP production is maximized. The exponential growth phase covers only the first hours until nitrogen exhaustion. In this phase, cells maximize growth. During growth under nitrogen limitation cells, still maximizing growth, accumulate carbohydrates. Thenceforward, a substantial fraction of the sugar is consumed during the stationary and decay phases by quiescent cells, which adjust their metabolism to cope with environmental fluctuations. In the last two phases, we assumed that cells maximize both ATP and protein production.

The general formulation of the FBA problem reads as follows:

$$\begin{array}{l} \underset{v}{\text{maximize}}\;J_{p}\\ \text{subject to}\\ \;\;\;\;\;\;\;\;\;\;\;\;\;\;\;\;\;\;\;\;\;\;\;\;S\cdot v=0\\ \;\;\;\;\;\;\;\;\;\;\;\;\;\;\;\;\;\;\;\;\;\;\;v_{{\text{NH}}_{\text{4}}}\;\geq \;f_{{\text{NH}}_{\text{4}}}\left({\text{NH}}_{\text{4}}\right)\\ \;\;\;\;\;\;\;\;\;\;\;\;\;\;\;\;\;\;\;\;\;\;\;v_{{\text{AA}}_{i}}\;\geq \;f_{\text{AA}}\left({\text{AA}}_{i}\right);\;\forall i=1,\cdots ,20\\ \;\;\;\;\;\;\;\;\;\;\;\;\;\;\;\;\;\;\;\;\;\;\;v_{\text{Glx}}=f_{\text{Glx}}\left(\text{Glx},E\right)\\ \;\;\;\;\;\;\;\;\;\;\;\;\;\;\;\;\;\;\;\;\;\;\;v_{F}=f_{F}\left(F,E\right)\\ \;\;\;\;\;\;\;\;\;\;\;\;\;\;\;\;\;\;\;\;\;\;v_{{\text{O}}_{2}}=f_{\text{O}}\left({\text{O}}_{2}\right)\\ \;\;\;\;\;\;\;\;\;\;\;\;\;\;\;\;\;\;\;\;\;\;\;v_{P_{j}}=f_{P_{j}}\left(v_{\text{Glx}},v_{F}\right);\;\forall j=1,\cdots ,20 \end{array}$$

where *J_p_* is the function to be maximized in each phase *p*, *S* is the stoichiometric matrix, *v* is the vector of fluxes in millimoles per gram (dry weight [DW]) per hour, *v_Glx_* and *v_F_* are the fluxes of glucose and fructose, *v_O_2__* is the flux of O_2_ present only at the beginning of the fermentation, *v*_NH_4__ is the flux of ammonium, *v*_AA_*i*__ is the exchange rate of the amino acid *i* (covering all 20 amino acids), and *v_P_j__* are the constraints associated with the j=1,…,20 fermentation products considered. Glx, *F*, NH_4_, AA*_i_*, and *P_j_* correspond to the concentrations of glucose, fructose, ammonium, amino acids, and products, all expressed in millimoles per liter. Table S2 presents the specific formulation for each phase.

10.1128/mSystems.00260-21.5 TABLE S2 Detailed mathematical formulation (objective and constraints) of the multiphase multiobjective pFBA implementation. Download Table S2, XLSX file, 0.08 MB. Copyright © 2021 Henriques et al. 2021 Henriques et al. https://creativecommons.org/licenses/by/4.0/ This content is distributed under the terms of the Creative Commons Attribution 4.0 International license.

The uptake of glucose and fructose was modeled using Michaelis-Menten (MM)-type kinetics with competitive ethanol inhibition (14):

(1) $$v_{\text{Glx}}=-v_{{\text{max}}_{G}}\cdot \frac{\text{Glx}}{\text{Glx}\;+\;k_{G}}\cdot \frac{1}{1\;+\;E/K_{\mathrm{Ei}}}$$

where *v_max_G__* is the maximum uptake rate, *k_G_* is the MM constant, *K_Ei_* is the strength of ethanol inhibitory effect, and *E* is its concentration (mmol/liter). A similar expression, *v_F_*, exists for fructose (*F*). Additionally, in our case studies, the medium was supplemented with sucrose; thus, we included a mass action-type expression, characterized by the kinetic constant *k*_hydro_, describing its hydrolysis.

A certain amount of dissolved oxygen is present in the medium and consumed during the lag phase (see Fig. S3B in Text S2). Its uptake is determined as follows:

(2) $$v_{{\text{O}}_{\text{2}}}\;\geq \;-k_{{\text{O}}_{\text{2}}}\cdot {\text{O}}_{2}\;$$

where *v_O_2__* and *k_O_2__* are the oxygen uptake and transport rate constants and O_2_ is the concentration of oxygen in the medium.

10.1128/mSystems.00260-21.2 TEXT S2 Bootstrap parameter estimation of the model. The text presents further numerical results, such as the convergence curves of the bootstrap approach, the distribution of parameter values, and the plots of the model versus data for all measured states not included in the main figures (amino acids, carbon, nitrogen and O_2_, carboxylic acids, esters, and higher alcohols). Download Text S2, PDF file, 2.8 MB. Copyright © 2021 Henriques et al. 2021 Henriques et al. https://creativecommons.org/licenses/by/4.0/ This content is distributed under the terms of the Creative Commons Attribution 4.0 International license.

The uptake of ammonium was modeled by

(3) $$v_{{\text{NH}}_{4}}\;\geq \;-v_{{\text{max}}_{{\text{NH}}_{\text{4}}}}\cdot \frac{{\text{NH}}_{4}}{{\text{NH}}_{4}\;+\;k_{{\text{NH}}_{4}}}$$

where NH_4_ is the extracellular concentration of ammonia (in millimoles per liter), *v*_max_NH_4___ is the maximum uptake rate achieved, and *k*_NH_4__ is the MM constant. To avoid an excessive number of parameters, amino acid transport was modeled following mass action kinetics:

(4) $$v_{{\text{AA}}_{i}}\;\geq \;-k_{{\text{AA}}_{i}}\cdot {\text{AA}}_{i}$$

where AA*_i_* is the extracellular concentration of the amino acid (in millimoles per liter) and *k*_AA_*i*__ is the associated kinetic parameter.

Production of alcohols and higher alcohols, carboxylic acids, and esters follows mass action kinetics:

(5) $$\dot{P_{i}}=X_{A}\cdot v_{P_{i}}$$

with *X_A_* the active biomass and the flux, *v_P_i__* , proportional to the amount of transported hexoses:

(6) $$v_{P_{i}}=-k_{P_{i}}\cdot \left(v_{\text{Glx}}+v_{F}\right)$$

where *P_i_* refers to the excreted product i=1,…,20 and *k_P_i__* the production rates.

An exception to this was the formulation of the dynamics of acetate. This metabolite is produced during exponential growth and consumed during stationary phase following mass action kinetics.

### Model of protein turnover.

Since nitrogen sources are depleted before the stationary phase, we developed a new model of nitrogen homeostasis that considered protein turnover. The proposed model describes the combined use of the Ehrlich and *de novo* synthesis pathways during stationary and decay phases to guarantee optimal adaptation to perturbations in nitrogen homeostasis.

To introduce protein turnover, we simulated the degradation of the existing protein fraction inside biomass ( Prot ), into a pool of amino acids that subsequently produce new proteins. During stationary and decay phases, the lower bounds on the amino acid uptake are set as

(7) $$v_{{\text{AA}}_{i}}\;\geq \;-\;\text{λ}\cdot \text{Prot}\cdot {\text{α}}_{{\text{AA}}_{i}}$$

where λ is the turnover rate, Prot is the concentration of protein, and α_AA*_i_*_ is associated with the stoichiometric coefficient of the amino acid *i* in the protein pseudoreaction. Mathematically, the dynamics of protein content reads as follows:

(8) $$\frac{d\text{Prot}}{\mathrm{dt}}=\text{μ}\cdot X_{v}\cdot {\text{Prot}}_{\text{content}}\;-\;\text{Prot}\cdot k_{\text{death}}\;+\;X_{A}\cdot v_{\text{Prot}}\;-\;\text{λ}\cdot \text{Prot}$$

where μ is the growth rate, Prot_content_ is the fraction of protein in the newly formed biomass, *k*_death_ (per hour) is the rate of biomass degradation during decay phase, *X_V_* is the simulated viable biomass (grams per liter), *X_A_* is the simulated active biomass (grams per liter), *v*_Prot_ is the protein production rate (grams per gram [DW] per hour), and λ is the protein turnover rate (per hour).

The degraded proteins ( λ·Prot ) are converted into extracellular amino acids whose concentrations are represented by the following equations:

(9) $$\frac{d{\text{AA}}_{i}}{\mathrm{dt}}=X\cdot v_{{\text{AA}}_{i}}+{\text{λ·Prot·α}}_{{\text{AA}}_{i}}$$

The rates *v*_AA_i__ are computed by maximizing protein production ( *v*_Prot_ ) and ATP ( *V*_ATP_ ) while solving the FBA problem:

$$\begin{array}{rrr} & \underset{v}{\text{maximize}} & v_{\text{Prot}},\phi \cdot v_{\text{ATP}}\\ & \text{subject to} & \\ & & S\cdot v=0\\ & & v_{\text{AA}}\;<\;\text{λ}\cdot \mathrm{Pr}{\text{ot·α}}_{\text{AA}}\\ & & v_{\text{Pro}}=\left(v_{\text{Glx}}\;+\;v_{F}\right)\cdot {\text{α}}_{\text{Pro}}\\ & & \ldots \end{array}$$

where *ϕ* is estimated for each strain, *S* is the stoichiometric matrix, *v* is the vector of fluxes, *v*_AA*_i_*_ is the exchange rate of amino acid AA*_i_*, *v_P_i__* are constraints associated with fermentation products, and *v*_Pro_ (*r_*1904) is the amount of excreted proline. The last amino acid accumulates in the extracellular medium throughout the fermentation (see Fig. S3 in Text S2), likely as a consequence of stored arginine consumption under anaerobic conditions (27). The extracellular dynamics of proline is described as follows:

(10) $$\frac{d\text{Pro}}{\mathrm{dt}}=X_{A}\cdot v_{\text{Pro}}$$

Depending on the kinetic constraints, amino acids can be directly incorporated into proteins or degraded to recover nitrogen for protein production. We observed that those amino acids which lacked pathways for their catabolism or elimination accumulated in the extracellular compartment during stationary phase. As an example, Fig. 1C1 shows how including a catabolic route for methionine, during stationary phase, successfully describes its dynamics, possibly indicating that this route could be active during the stationary phase.

### Dynamic biomass equation.

A static biomass equation was not able to explain nitrogen assimilation (Fig. 1C2). Therefore, we implemented the following dynamic biomass equation (15):

(11) $${\text{Prot}}_{\text{content}}=A\cdot \left(1\;-\;e^{B\cdot \text{YAN}}\right)$$

where *A* and *B* are estimated parameters and yeast assimilable nitrogen (YAN) accounts for the ammonium and free amino acids present in the medium, excluding proline, which is not catabolized under anaerobic conditions.

Furthermore, we assumed that mRNA level was proportional to the protein content ( mRNA = Prot/RNA-to-Prot ratio ). In this framework, carbohydrates compensate for the variation in protein and mRNA content. Growth-associated ATP maintenance (GAM) was also updated to account for the polymerization costs of the different macromolecules (protein, RNA, DNA, and carbohydrates):

(12) $${\text{GAM = GAM}}_{\text{fitted}}{\;+\;\text{GAM}}_{\text{Prot}}{\;+\;\text{GAM}}_{\text{RNA}}{\;+\;\text{GAM}}_{\text{Carbs}}\;+\;{\text{GAM}}_{\text{DNA}}\;$$

where GAM_fitted_ is a species- or strain-dependent parameter estimated from data and the rest are polymerization costs of the different biomass precursors (adapted from reference 18). Additionally, to represent the premature end of fermentations during the decay phase (observed in SuCECT12600), we estimated the non-growth-associated maintenance (NGAM).

In addition, we discriminated between active cells (able to ferment) and viable cells (able to divide and ferment) to capture the dynamics of CFU and biomass (Fig. 1C2). The dynamics of active cell mass is represented by the equation

(13) $$\dot{X_{A}}=\text{μ}\cdot X_{V}\;-\;X_{A}\cdot k_{\text{decay}}\;+\;X_{A}\cdot v_{\text{Prot}}\;-\;\text{λ·Prot}$$

where *X_A_* is the active cell mass (grams per liter), μ is the growth rate computed with pFBA, *k*_decay_ is the decay rate (only active during decay), *v*_Prot_ (*r_*4047) is the exchange flux for protein production, and λ is the turnover rate (both only active during stationary and decay phases). The behavior of viable cell mass differed from that of active cell mass by a decline induced by ethanol (28):

(14) $$\dot{X_{v}}=\dot{X_{A}}-X_{v}\cdot k_{E\text{death}}\cdot \frac{E^{n}}{E^{n}\;+\;k^{n}}$$

where *X_v_* (grams per liter) is the growth rate obtained with the constraint based model, *E* is the ethanol concentration (millimoles per liter), and *k*_*E*death_ , *n*, and *k* are the parameters controlling susceptibility to ethanol.

The former mechanisms, coupled to parameter estimation, allowed us to predict nitrogen consumption, CFU, and biomass dynamics accurately (Fig. 1C3 to Fig. 1C6).

### Goodness of fit of the model in the case studies.

**FIG 2:**
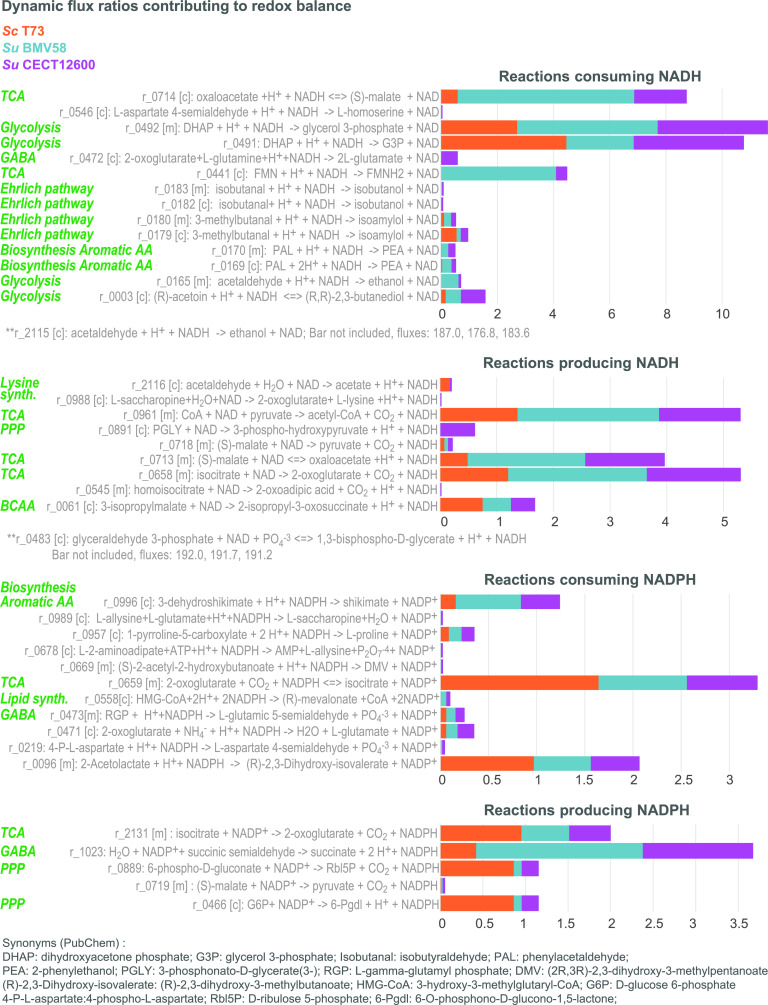
Comparative study of the fluxes through the reactions consuming and producing NADH and NADPH at the stationary phase, illustrating how strains achieve redox balance. The most significant differences between strains are found at the level of the TCA cycle, the GABA shunt, the pentose phosphate pathway, the biosynthesis of aromatic amino acids which eventually lead to production of 2-phenylethanol (PEA) and the Ehrlich pathway toward production of isoamylol. It is also important to note that S. uvarum diverts flux to the production of mevalonate.

**FIG 3:**
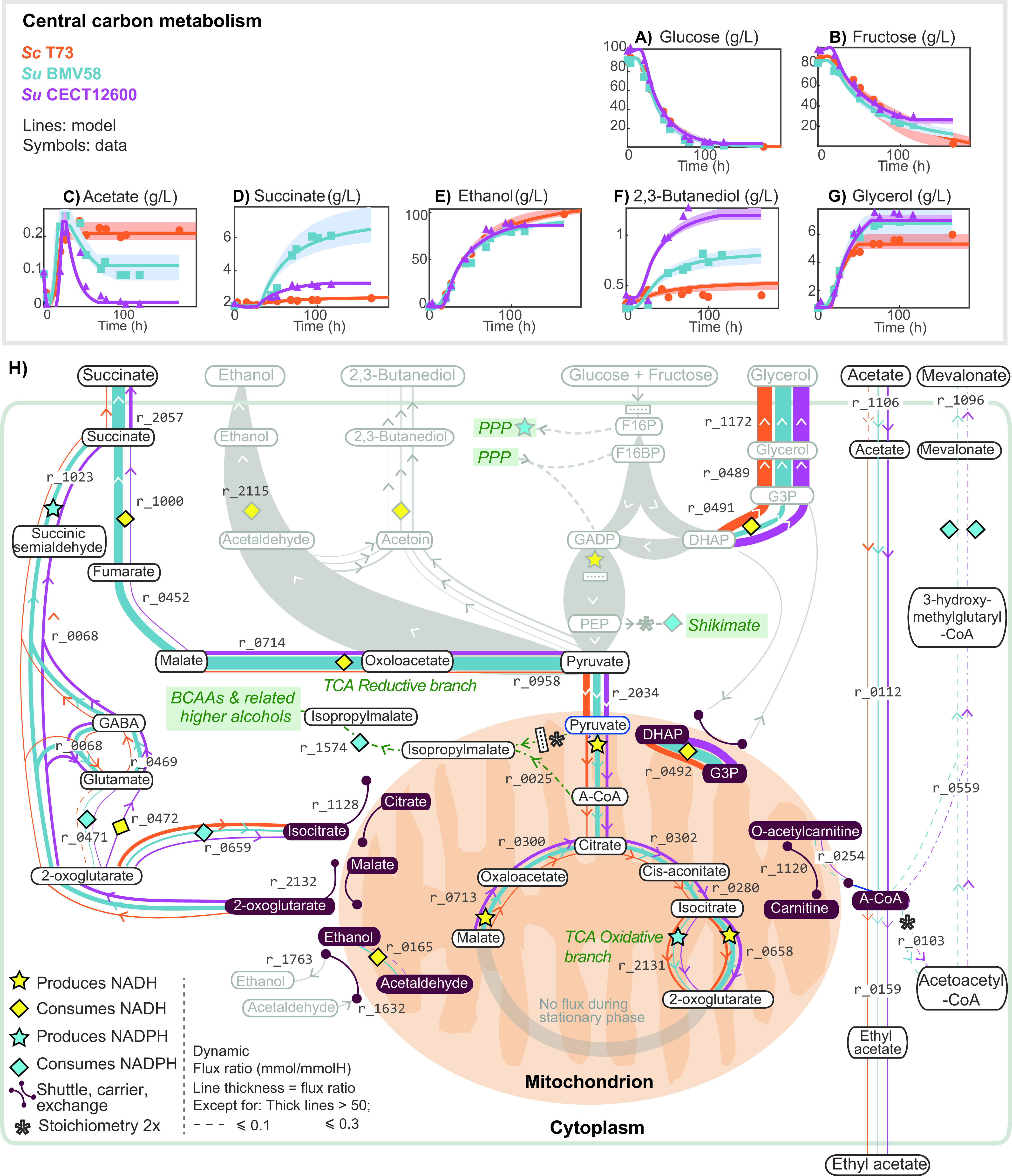
Redox balance in central carbon metabolism. Panels A to F show model predictions versus the experimental data extracellular metabolite concentrations associated with glycolysis and central carbon metabolism for the three strains. Panel H presents the predicted intracellular dynamic flux ratios during the stationary phase, showing how S. uvarum and S. cerevisiae strains use different redox balance strategies. These differences result in the differential production of relevant external metabolites such as acetate (C), succinate (D), ethanol (E), 2,3-butanediol (F), and glycerol (G). Explicit differences in the pathways in gray are presented in Text S3; otherwise, as indicated in the legend, width of the lines is proportional to the dynamic flux ratio.

**FIG 4:**
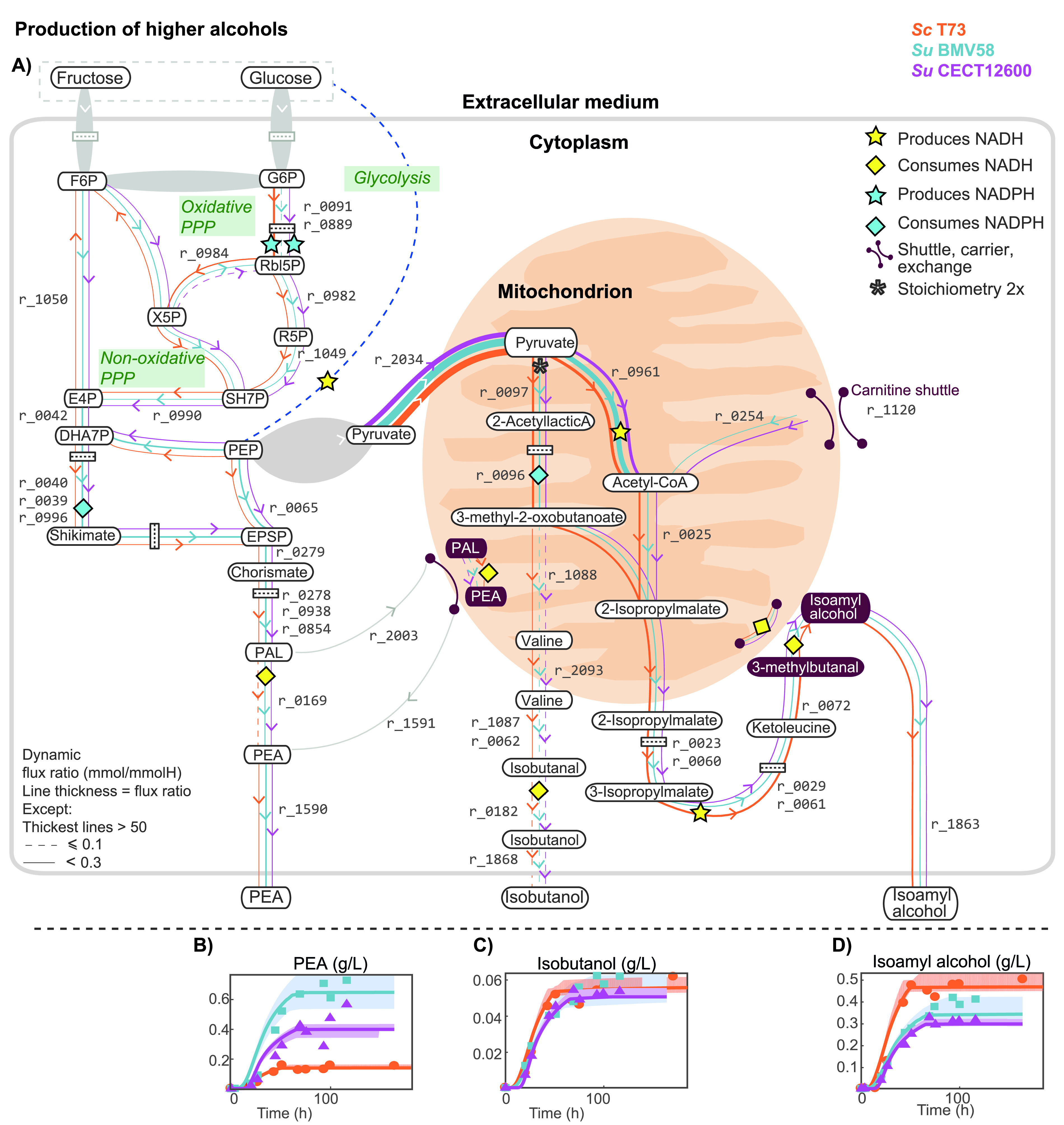
Redox balance in higher-alcohol production. Panel A shows the predicted intracellular flux ratios (above 0.01 mmol/mmolH) related to higher alcohols 2-phenylethanol (PEA), isobutanol, and isoamyl alcohol during the stationary phase and their corresponding impact on the redox balance of cofactors NADPH/NADP^+^ and NADH/NAD^+^. Panels B to D correspond to the comparison between model predictions and raw measures of PEA, isobutanol, and isoamyl alcohol, respectively. Other higher alcohols, such as methionol and tyrosol, seemed to accumulate in minimal quantities (flux ratio 100≃0.001 ) in response to perturbations in the amino acid pool.

The final model consisted of 46 ordinary differential equations depending on 66 parameters which we estimated from time series data for all measured external metabolites and biomass. The mean standard deviation on the parameters ranges from 2.5% for SuCECT12600 and 12.6% for SuBMV58. The reasonably low distribution on the parameters resulted in a reasonably low uncertainty associated with the model simulations (as seen in Fig. 2, 3, and 4).

10.1128/mSystems.00260-21.3 TEXT S3 Detailed description of the redox balance mechanisms used by the different strains. The text discusses in detail the production of ethanol, glycerol, succinate, 2,3-butanediol, and higher alcohols, production and consumption of acetate, and contributions to the redox balance. Download Text S3, PDF file, 1.1 MB. Copyright © 2021 Henriques et al. 2021 Henriques et al. https://creativecommons.org/licenses/by/4.0/ This content is distributed under the terms of the Creative Commons Attribution 4.0 International license.

The model described the dynamics of our illustrative examples successfully. The best fit to the data and the associated uncertainty, as computed by the bootstrap, are shown in Text S2 and Fig. 3 and 4. We determined the *R*-squared measure of goodness of fit ( *R*^2^ ) for each measured variable and each strain-based fermentation. The medians of the *R*^2^ values are above 0.94 for all strains.

Interested readers may find further details on the parameter estimation in Text S2. Optimal parameter values and *R*^2^ values are reported in Table S3.

10.1128/mSystems.00260-21.6 TABLE S3 Optimal parameter values and goodness of fit of the model. Table sheet 1 presents the list of all parameters together with their optimal value as achieved by the bootstrap approach for the three individual strains. The comparison between the wine strains ScT73 and SuBMV58 reveals substantial differences—above 100% relative difference—in the growth-associated ATP maintenance, the rate of transport of specific amino acids and hexoses, and the rate of production of succinate. The lag phase is more than three times longer for the natural strain; in addition, the non-growth-associated maintenance is practically zero for wine strains, while it is around 0.8 for the SuCECT12600 strain. The uptake of various amino acids varies significantly between S. uvarum strains. In particular, the uptake rate for threonine is 740% higher for SuCECT12600 than for SuBMV58. Table sheet 2 presents the *R*^2^ goodness-of-fit as computed for all measured compounds and biomass for the three individual strains. The vast majority of the coefficients were above 0.9. Some negative values appeared—10 out of 141—typically associated with low signal-to-noise ratio and high data dispersion observed in the measured variable, e.g., cysteine. Download Table S3, XLSX file, 0.02 MB. Copyright © 2021 Henriques et al. 2021 Henriques et al. https://creativecommons.org/licenses/by/4.0/ This content is distributed under the terms of the Creative Commons Attribution 4.0 International license.

### Species behavior differs significantly in the stationary phase.

At the extracellular level, the most striking differences between strains occur in the production of compounds associated with the central carbon metabolism and nitrogen metabolism, in particular, in the dynamics of acetate and the yields of succinate, 2,3-butanediol, and glycerol (Fig. 3C, D, F, and G) and in the production of 2-phenylethanol and isoamyl alcohol (Fig. 4B and D).

We used the model to decipher the metabolic strategies used by the different strains that lead to such differences. Table S4 reports the dynamic flux ratios computed for the overall process and the different phases using equations 21 to 23 for those reactions in which the maximum flux ratio value over the three species is above 0.01 mmol/mmolH (where mmolH is millimoles of consumed hexose × 100). Uncertainties associated with the fluxes due to uncertainties on the parameters are also reported.

10.1128/mSystems.00260-21.7 TABLE S4 Dynamic flux ratios and transcriptomic data. Sheets 1 to 4 present the overall, growth, decay, and stationary dynamic flux ratios, respectively, as millimoles of produced compound per millimole of consumed hexose × 100 (mmol/mmolH). Only those reactions for which the maximum flux ratio value over the three species is above 0.01 mmol/mmolH are reported. Sheet 4 presents the unpublished data by M. López-Malo, A. Querol, and J. M. Guillamon showing substantial accumulation of GABA in S. uvarum strains. Download Table S4, XLSX file, 0.2 MB. Copyright © 2021 Henriques et al. 2021 Henriques et al. https://creativecommons.org/licenses/by/4.0/ This content is distributed under the terms of the Creative Commons Attribution 4.0 International license.

Text S3 summarizes the differences observed in flux ratios between species and phases. Again, the intracellular behavior differs significantly in the stationary phase. Thus, subsequent sections elaborate on the metabolic study of the stationary phase. Remarkably, this is also the phase in which higher alcohols and aroma are produced, thus being relevant for industrial applications.

### The GABA shunt as an NADPH source in cryotolerant species.

Cells produced the most significant fraction of succinate during the stationary and decay phases, with a significantly higher dynamic flux ratio by SuBMV58 and SuCECT1600 ( 6.08 and 1.66) than ScT73 ( 0.42, *r*_2057 ).

During the decay phase, most succinate was produced through the tricarboxylic acid (TCA) cycle reductive branch in the two species. However, during the stationary phase, succinate production was distributed between the GABA shunt (ScT73, 0.42; SuBMV58, 2.00; SuCECT12600, 1.31; Fig. 3H, *r*_1023 ) and the reductive branch of TCA (ScT73, 0.00; SuBMV58, 4.10; and SuCECT12600, 0.35; Fig. 3H, *r*_1000 ). Remarkably, succinate production through the GABA shunt was between 3 and 4.6 times higher for S. uvarum species than for S. cerevisiae (Fig. 3H, *r*_0068 , *r*_1023 ). This result suggested an important role for the GABA shunt in the maintenance of the cellular redox state. Incidentally, revisiting data from reference 29, we found a considerable accumulation of GABA ( 93.59-fold change) by SuCECT12600 (Table S4).

### Intracellular mevalonate as a reducing equivalent in cryotolerant yeast species.

The three strains produced acetate during the growth phase (ScT73, 1.079; SuBMV58, 1.145; SuCECT12600, 1.422 [Table S4, *r*_1106 ]) and until the entry into the stationary phase. Afterward, while the extracellular acetate concentration remained constant in ScT73, a decrease was observed in both S. uvarum fermentations, indicating acetate consumption. As shown in Fig. 3C, our model successfully described these phenotypes.

According to the modeling constraints, the most parsimonious explanation for this observation would have been an operative glyoxylate cycle. However, based on the repression by glucose of the key enzymes of the glyoxylate cycle (i.e., ICL1 and MLS1) and previous intracellular data (29), we decided to block this cycle and explore an alternative hypothesis. As a result, the model suggested that S. uvarum strains incorporated the acetate derivative, acetyl coenzyme A (acetyl-CoA), into mevalonate (SuBMV58, 0.19, and SuCECT12600, 0.10; *r*_0559 and *r*_0103 ) also consuming NADPH. In addition, mevalonate is a reducing equivalent that can be further metabolized into, for example, ergosterol ( *r*_0127 in our reconstruction) possibly acting as storage of NADPH in cryotolerant species.

S. uvarum strains also used the carnitine shuttle system to transport acetyl-CoA into the mitochondria (SuBMV58, 0.09, and SuCECT12600, 0.38; Fig. 3H, *r*_0254 ). Inside the mitochondria, acetyl-CoA was used to form isopropylmalate (Fig. 4A)—a precursor of leucine and isoamyl alcohol—or in the TCA oxidative branch toward the synthesis of 2-oxoglutarate (Fig. 3H).

### The production of higher alcohols contributed to the redox balance.

Higher-alcohol production was most prominent during the stationary phase for the three strains. Our model predicted that carbon skeletons of isoamyl alcohol, 2-phenylethanol (PEA), and isobutanol were in great part synthesized *de novo* from glycolytic and pentose phosphate pathway (PPP) intermediates, rather than coming from the catabolism of precursor amino acids (leucine, valine, and phenylalanine, respectively) (Fig. 4A).

S. uvarum strains produced more PEA than ScT73 (ScT73, 0.158; SuBMV58, 0.659; and SuCECT12600, 0.383; *r*_1590 ), while the opposite occurred for isoamyl alcohol (ScT73, 0.736; SuBMV58, 0.483; and SuCECT12600, 0.394; *r*_1863 ). We found that the production of PEA and isoamyl alcohol contributed substantially to the redox metabolism related to glycerol accumulation. Approximately 43% , 36% , and 27% of the glycerol produced by the ScT73, SuBMV58, and SuCECT12600 strains was attributable to NADH derived from isoamyl alcohol and PEA.

The higher production of PEA observed in S. uvarum strains occurs through the higher flux through the shikimate pathway (Fig. 4A, *r*_0996 and *r*_0279 ). Interestingly, while ScT73 had a larger flux ratio through the oxidative pentose phosphate pathway (Fig. 4A, *r*_0091 and *r*_0889 ) partly redirected toward glycolysis, S. uvarum simulations reflected the inverse pattern (Fig. 4A, *r*_0984 ), with glycolytic flux being shifted toward the nonoxidative PPP.

Pyruvate in the mitochondrion showed two different fates: acetyl-CoA ( *r*_0961 ) and 2-acetyllactic acid ( *r*_0097 ). Noticeably, S. uvarum strains also contributed to acetyl-CoA using the carnitine shuttle ( *r*_0254 ). 2-Acetyllactic acid can further be converted to 3-methyl-2-oxobutanoate, consuming one NADPH ( *r*_0096 ), which also showed two different fates: the production of 2-isopropylmalate (leading to isoamyl alcohol; *r*_0025 , *r*_0072 , and *r*_0179 ) or valine (leading to the synthesis of isobutanol via the Ehrlich pathway; *r*_1087 , *r*_0062 , and *r*_0182 ).

Production of PEA and isoamyl alcohol also affected NADP^+^/NADPH metabolism. While the GABA shunt and oxidative PPP were the main producers of NADPH, consumption of NADPH was attributable mostly to isoamyl alcohol and PEA. In fact, the increased isocitrate dehydrogenase flux ( *r*_0659 ) observed in ScT73 ( 1.646 against 0.917 and 0.727 in the S. uvarum strains) was associated with the need for shuttling NADPH into the mitochondria ( *r*_2131 ), utilized in 3-methyl-2-oxobutanoate synthesis (precursor of the isoamyl alcohol; see Fig. 3H and Fig. 4A). On the other hand, production of PEA without the oxidative PPP (predicted in the S. uvarum strains) resulted in excess NADP+. In the case of S. uvarum, given the reduced influence of oxidative PPP, NADP^+^ recycling was achieved mostly through the GABA shunt.

## DISCUSSION

Genome-scale models have the potential to decipher how nonconventional yeast species use metabolism to produce industrially relevant products and tolerate specific stressors, such as cold temperatures. This study aimed to develop a dynamic genome-scale model to investigate the dynamics of yeasts primary and secondary metabolism in batch cultures. To generate biological hypotheses for modeling, we considered the description of the metabolism of S. cerevisiae and cryotolerant S. uvarum strains in a rich medium (grape must) fermentation.

The first question in this research was how to model all phases in the batch process: lag, exponential growth, limited nitrogen growth, stationary, and decay. Prior studies focused on the exponential growth phase and were based on available reconstructions with many missing reactions, particularly those related to secondary metabolism (14, 15, 17, 30). Also, their static nature hinders the description of the sequential nature of amino acid consumption (31).

As a first step, we needed to extend a yeast genome-scale reconstruction to account for the production of higher alcohols, carboxylic acids, or esters. We extended the Yeast8 consensus model incorporating missing reactions and metabolites. A similar curation process has been recently applied to the iMM904 reconstruction (32). The authors fitted the model to data from the literature, concluding that further curation and adaptations were necessary to successfully predict metabolism and biomass dynamics. We experienced such difficulties in our first iterations in the modeling process and introduced several new features to obtain more accurate simulations of carbon and nitrogen metabolism throughout time.

A critical aspect for an improved accuracy was the multiphase multiobjective dynamic FBA scheme. Previous works focused on ATP consumption to explain the metabolism after depletion of the limiting nutrient (15, 33, 34); we incorporated the production of protein as a cellular objective (together with protein degradation) with accurate results. Also, we modeled protein turnover to account for the uptake of amino acids and inorganic nitrogen in the stationary phase. To the best of our knowledge, this is the first dynamic genome-scale metabolic model describing nitrogen homeostasis during the stationary phase. Finally, we introduced a dynamic biomass equation which further improved the model accuracy. This result agrees with observations in previous studies (35–37) pointing out the relevance of detailing biomass composition in a context-specific manner.

The second question in this research was to decipher the differences in the metabolism of three strains of two different species, S. cerevisiae and S. uvarum, in rich medium (grape must) fermentation. Recently, it was hypothesized that S. cerevisiae and S. uvarum species might have different redox balance strategies (38). Notably, the model confirmed this hypothesis and brought novel insights into the specific routes used by the two species.

Our predictions suggest alternative pathways for cryotolerant species to produce succinate and consume acetate. In principle, yeasts might form succinate via four main pathways, all based on the reactions of the TCA cycle (39). The selected pathway depends on the environmental conditions and strain. Our model predicted that ScT73 and SuBMW58 produced overall the most succinate via the TCA reductive branch, in agreement with previous findings (40). However, our results also suggest an important role for the TCA oxidative branch until 2-oxoglutarate for the S. uvarum strains during the stationary phase. This result is consistent with the recent intracellular data obtained by Minebois et al. (25), who observed a noticeable intracellular accumulation of 2-oxoglutarate in SuBMV58.

One somewhat unexpected finding of the model was the extent to which the GABA shunt would contribute to succinate formation during the stationary phase, an effect particularly evident in the case of S. uvarum strains. The role of this pathway in yeast is not fully understood (41–43). Bach et al. (41) observed that glutamate decarboxylase (encoded by *GAD1*) was poorly expressed when succinate was produced in S. cerevisiae and that the GABA shunt played a minor role in redox metabolism. In contrast, Coleman et al. (44) showed that *GAD1* expression is required for oxidative stress tolerance in S. cerevisiae. Similarly, Cao et al. (42) showed that *GAD1* confers resistance to a heat stress effect that might be related to NADPH production. Additionally, *GAD1* was upregulated during the stationary phase under nitrogen starvation (41, 43, 45), and López-Malo et al. (29) observed high intracellular GABA levels in the cryotolerant species S. uvarum.

Recently, Liu et al. (46) found clear indication that the GABA shunt may be involved in supplying NADPH for lipid synthesis in the oleaginous yeast Yarrowia lipolytica. Also, Bach et al. (41) showed that S. cerevisiae can degrade GABA into succinate or γ-hydroxybutyric acid (GHB) and that GHB was used to form the polymer polyhydroxybutyrate (PHB). Also noteworthy is that PHBs are synthesized by numerous bacteria as carbon and energy storage compounds (47). PHBs are also strongly associated with bacterial cold tolerance (48), suggesting a similar function in yeast.

Another important finding is that S. uvarum strains consume acetate once nitrogen sources are depleted, coinciding with the extracellular accumulation of succinate. This finding was also reported by Kelly et al. (49), who showed that an S. uvarum yeast isolate can metabolize acetate, resulting in lower acetic acid, ethyl acetate, and acetaldehyde concentrations in wine. The model predicted that some of the acetate carbon was directed toward mevalonate, which is in line with recent experimental work by Minebois et al. (25). According to Bach et al. (41), a route for acetyl-CoA incorporation into PHB polyester through 3-hydroxybutyrate-CoA seems plausible. However, this hypothesis is not taken into account by the genome-scale reconstructions.

The fact that López-Malo et al. (29) found high intracellular GABA and GHB in cryotolerant strains grown in synthetic must (without GABA) at low temperature, and the flux predicted in the present work, suggests that S. uvarum stores lipids or polyesters (i.e., PHBs) as reducing equivalents to withstand oxidative stress induced by low temperatures. The role of the GABA shunt and the production of reducing equivalents in the metabolism of cryotolerant species may be plausible routes worth exploring.

Our model also predicted that the carbon skeletons of higher alcohols (e.g., isobutanol and isoamyl alcohol) were mainly synthesized *de novo* rather than from the incorporation and catabolism of amino acids (e.g., leucine and valine). This result agrees with the findings of Crépin et al. (50), who explored the fate of the carbon backbones of aroma-related exogenous amino acids using ^13^C isotopic tracer experiments. Similarly, our results indicate that 2-phenylethanol was mostly synthesized *de novo*. We hypothesize that a positive contribution in glycerol content may also explain why the production of 2-phenylethanol and isoamyl alcohol is a conserved evolutionary trait in yeasts.

Noticeably, most of NADPH consumption was associated with higher-alcohol synthesis. The increased flux through cytosolic isocitrate dehydrogenase associated with isoamyl alcohol is compatible with reports associating high expression of IDP2 with nitrogen deficiency (51) and stationary phase (52). Furthermore, the prediction that during the stationary phase, PEA is produced through the chorismate synthesis pathway (downstream of the nonoxidative PPP), thus regenerating some NADPH associated with the conversion of succinic semialdehyde into succinate, provides a counterintuitive rationale for understanding the correlation between the observed differences in higher-alcohol and succinate production between S. cerevisiae and S. uvarum.

Interestingly, the model predicts that most other higher alcohols (tyrosol, methionol, etc.) accumulate in small amounts due to perturbations in the amino acid pool. These results confirm the hypothesis raised by Shopska et al. (53), who suggested that the two schemes to produce higher alcohols—the Ehrlich pathway and *de novo* synthesis—are not in contradiction but two extremes of a common mechanism. Incidentally, Yuan et al. (54) showed that the assembled leucine biosynthetic pathway coupled with the Ehrlich degradation pathway results in high-level production of isoamyl alcohol.

Our bootstrap-based identifiability analysis of the proposed model and the fact that model predictions are consistent with numerous previous findings lead us to conclude that the present model (along with the provided code) can simulate yeast metabolism in batch culture in a general chemically characterized medium; the only requirement would be to update the metabolic reconstruction if required for the specific yeast species. The model can also be used to explore and engineer novel metabolic pathways toward specific bioproducts.

## MATERIALS AND METHODS

### Yeast strains.

In this study, three yeast strains belonging to S. cerevisiae and S. uvarum were used: the commercial strain T73 (Lalvin T73 from Lallemand Montreal, Canada), originally isolated from wine in Alicante, Spain (55), was selected as our wine S. cerevisiae (ScT73) representative; the commercial strain BMV58 (SuBMV58, Velluto BMV58 from Lallemand Montreal, Canada), originally isolated from wine in Utiel-Requena (Spain), and the noncommercial CECT12600 strain, isolated from a nonfermentative environment (SuCECT12600, Alicante, Spain), represented S. uvarum.

### Fermentation experiments.

Fermentation assays were performed with grape must obtained from Merseguera white grapes, collected in the 2015 vintage in Titaguas (Spain) and stored in several small frozen volumes (4 liters at 20°C). Before its use, the must was clarified by sedimentation for 24 h at 4°C and sterilized by adding dimethyl dicarbonate at 1 ml liter^−1^. All fermentations were performed in 3 independent biological replicates in 500-ml controlled bioreactors (MiniBio; Applikon, The Netherlands) filled with 470 ml of natural grape must. Each bioreactor was inoculated using an overnight starter culture cultivated in Erlenmeyer flasks containing 25 ml of YPD medium ( 2% glucose, 0.5% peptone, 0.5% yeast extract) at 25°C and 120 rpm in an agitated incubator (Selecta, Barcelona, Spain). Strain inoculation was done at an optical density at 600 nm (OD_600_) of 0.100. The dynamics of the fermentation was registered using different probes and detectors to control and measure temperature, pH, dissolved oxygen (Applikon, The Netherlands), and effluent carbon dioxide level (INNOVA 1316 multigas monitors; LumaSense Technologies). Data were integrated into the BioExpert software tools (Applikon, The Netherlands). The fermentation was complete when a constant sugar content was reached as measured by high-performance liquid chromatography (HPLC).

### Sampling and quantification of extracellular metabolites.

Extracellular metabolites, including sugars, organic acids, main fermentative by-products, and yeast assimilable nitrogen (YAN), were determined at 10 sampling times during the fermentation. Residual sugars (glucose and fructose), organic acids (acetate, succinate, citrate, malate, and tartrate), and the main fermentative by-products (ethanol, glycerol, and 2,3-butanediol) were quantified using HPLC (Thermo Fisher Scientific, Waltham, MA) coupled with refraction index and UV-visible (UV-Vis) (210-nm) detectors. Metabolites were separated through a HyperREZ XP Carbohydrate H+ 8-μm column coupled with a HyperREZ XP carbohydrate guard (Thermo Fisher Scientific). The analysis conditions were as follows: eluent, 1.5 mM; flux, 0.6 ml min^−1^; and oven temperature, 50°C. For sucrose determination, the same HPLC was equipped with a Hi-Plex Pb, 300- by 7.7-mm column (Agilent Technologies, CA), and the following analysis conditions were used: eluent, Milli-Q water; flux, 0.6 ml min^−1^; and oven temperature, 50°C. The retention times of the eluted peaks were compared to those of commercial analytical standards (Sigma-Aldrich, Madrid, Spain). Metabolite concentrations were quantified by the calibration graphs (*R*^2^ value > 0.99) of the previously obtained standards from a linear curve fit of the peak areas using 10 standard mixtures.

Determination of yeast assimilable nitrogen in the form of amino acids and ammonia was carried out following the same protocol as described previously (56). A volume of supernatant was removed from the fermentor, and amino acids and ammonia were separated with an ultraperformance liquid chromatograph (UPLC; Dionex Ultimate 3000; Thermo Fisher Scientific, Waltham, MA) equipped with a Kinetex 2.6-μm C_18_ 100A column (Phenomenex, Torrance, CA) and Accucore C_18_ 10- by 4.6-mm, 2.6-μm Defender guards (Thermo Fisher Scientific, Waltham, MA). For derivatization, 400 μl of the sample was mixed with 430 μl of borate buffer (1 M, pH 10.2), 300 μl of absolute methanol, and 12 μl of diethyl ethoxymethylenemalonate (DEEMM) and ultrasonicated for 30 min at 20°C. The ultrasonicated sample was incubated up at 80°C for 2 h to allow the complete degradation of excess DEEMM. Once the derivatization finished, the sample was filtered with a 0.22-μm filter before injection. The target compounds in the sample were then identified and quantified according to the retention times, UV-Vis spectral characteristics, and calibration curves (*R*^2^ value > 0.99) of the derivatives of the corresponding standards. Amino acid standard (reference no. AAS18), asparagine, and glutamine purchased from Sigma-Aldrich were used for calibration.

### Higher alcohols and esters.

We also determined the concentrations of higher alcohols and esters for each sampling time. Volatile-compound extraction and gas chromatography (GC) were performed following the protocol described in reference 71. Extraction was performed using headspace solid-phase-microextraction sampling (SPME) with polydimethylsiloxane (PDMS) fibers (Supelco; Sigma-Aldrich, Barcelona, Spain). Aroma compounds were separated by GC in a Thermo TRACE GC Ultra chromatograph (Thermo Fisher Scientific, Waltham, MA) equipped with a flame ionization detector (FID), using an HP-INNOWAX 30-m by 0.25-mm capillary column coated with a 0.25-mm layer of cross-linked polyethylene glycol (Agilent Technologies, CA). Helium was the carrier gas used (flow, 1 ml min^−1^). The oven temperature program was as follows: 5 min at 60°C, 5°C min^−1^ to 190°C, 20°C min^−1^ to 250°C, and 2 min at 250°C. The detector temperature was 280°C, and the injector temperature was 220°C under splitless conditions. The internal standard was 2-heptanone (0.05% [wt/vol]). Volatile compounds were identified by the retention time for reference compounds. The quantification of the volatile compounds was determined using the calibration graphs of the corresponding standard volatile compounds.

### Physiological and biomass parameters.

Physiological and biomass parameters, including OD_600_, dry weight (DW), CFU, and average cell diameter (ACD), were determined at each sample time, provided that the cell sample was sufficient to perform the corresponding measure. DW determination was performed by centrifuging 2 ml of the fresh sample placed in a preweighed Eppendorf tube in a MiniSpin centrifuge (Eppendorf, Spain) at maximum speed (13,200 rpm) for 3 min. After centrifugation, the supernatant was carefully removed, and the pellet was washed with 70% (vol/vol) ethanol and centrifuged under the same conditions. After washing, the aqueous supernatant was removed carefully and the tube placed in a 65°C oven for 72 h. DW was finally obtained by measuring the mass weight difference of the tube with a BP121S analytical balance (Sartorius, Goettingen, Germany). OD_600_ was measured at each sampling time using a diluted volume of sample and a BioPhotometer spectrophotometer (Eppendorf, Germany). CFU were determined using 100 to 200 μl of a diluted volume of samples plated in YPD solid medium ( 2% glucose, 2% agar, 0.5% peptone, 0.5% yeast extract) and incubated for 2 days at 25°C. The resulting colonies were counted with a Comecta S.A. colony counter. Only plates with a CFU count between 30 and 300 were used to calculate the CFU of the original sample. For ACD determination, a volume of cell sample was diluted into a phosphate-buffered saline solution and cell diameter measured using a Scepter handheld automated cell counter equipped with a 40-μm sensor (Millipore, Billerica, MA).

### Orthology analysis and genome-scale metabolic reconstruction.

Genomes of ScT73 (57) and SuBMV58 and SuCECT12600 (PRJNA471597; M. Morard, L. G. Macías, A. C. Adam, M. Lairón-Peris, R. Pérez-Torrado, C. Toft, and E. Barrio, unpublished data) were sequenced and assembled previously. Genome assemblies were annotated by homology and gene synteny using RATT (58). This approach let us transfer the systematic gene names of S. cerevisiae S288c annotation (59) to our assemblies and, therefore, to select only those syntenic orthologous genes in the T73, CECT12600, and BMV58 genomes for subsequent analyses.

We added to the consensus genome-scale reconstruction of Saccharomyces cerevisiae S288C (v.8.3.2) metabolites and reactions related to amino acid degradation and higher-alcohol and ester formation. This refined model was then used as a template for reconstructing strain-specific genome-scale models for SuBMV58, SuCECT12600, and ScT73. MetaDraft, AuReMe and the results from the orthology analysis were used to create the strain-specific models.

### Flux balance analysis.

Flux balance analysis (FBA) (60, 61) is a modeling framework based on knowledge of reaction stoichiometry and mass/charge balances. The framework relies on the pseudo steady-state assumption (no intracellular accumulation of metabolites occurs). This is captured by the well-known expression

(15) S·v=0

where *S* is stoichiometric matrix of (*n* metabolites by *m* reactions) and *v* is a vector of metabolic fluxes. The number of unknown fluxes is higher than the number of equations, and thus, the system is undetermined. Still, it is possible to find a unique solution under the assumption that cell metabolism evolves to pursue a predetermined goal which is defined as the maximization (or minimization) of a certain objective function (*J*):

(16, 17, 18, 19) $$\begin{array}{rrr} & \text{max }J & \\ s.t.: & & \\ & S\cdot v=0 & \\ & \text{LB}\;<\;v\;<\;\text{UB} & \end{array}\;$$

where LB and UB correspond to the lower and upper bounds on the estimated fluxes. Examples of objective functions *J* include growth rate, ATP, and the negative of nutrient consumption, etc.

Typically, multiple optimal solutions exist for a given FBA problem. In parsimonious FBA (pFBA), the result is the most parsimonious of optimal solutions, i.e., the solution that achieves the specific objective with the minimal use of gene products and the minimization of the total flux load (62).

### Parameter estimation.

The aim of parameter estimation is to compute the unknown parameters—growth-related constants and kinetic parameters—that minimize some measure of the distance between the data and the model predictions. The maximum likelihood principle yields an appropriate measure of such distance (63):

(20) $$J_{\mathrm{mc}}\left(\text{θ}\right)=\overset{n_{\text{exp}}}{\underset{k=1}{\sum }}\overset{n_{\text{obs}}}{\underset{j=1}{\sum }}\overset{n_{\text{st}}}{\underset{i=1}{\sum }}{\left(\frac{y_{k,j,i}\left(\text{θ}\right)\;-\;y_{k,j,i}^{m}}{\sigma_{k,j,i}}\right)}^{2}\;$$

where *n*_exp_ , *n*_obs_ , and *n*_st_ are, respectively, the number of experiments, observables (measured quantities), and sampling times, while $\sigma_{k,j,i}$ represents the standard deviation of the measured data as obtained from the experimental replicates. *y_jm_* represents each of the measured quantities, *X^m^* and *C^m^* in our case, and $y_{j}\left(\text{θ}\right)$ corresponds to model predicted values, *X* and *C*. Observation functions were included for CFU and OD_600_ in order to scale viable cell mass (*X_v_*) and active cell mass (*X_A_*), respectively.

Parameters are estimated by solving a nonlinear optimization problem where the aim is to find the unknown parameter values (θ) to minimize $J_{\mathrm{mc}}\left(\text{θ}\right)$ , subject to the system dynamics—the model—and parameter bounds (64).

### Uncertainty analysis.

In practice, the value of the parameters θ compatible with noisy experimental data is not unique; i.e., parameters are affected by some uncertainty (64). The consequence of significant parametric uncertainty is that it may impact the accuracy of model predictions.

To account for model uncertainty, we used an ensemble approach. To derive the ensemble, we apply the bootstrap smoothing technique, also known as bootstrap aggregation (the bagging method) (65, 66). The bagging method is a well-established and effective ensemble model/model averaging device that reduces the variability of unstable estimators or classifiers (66). The underlying idea is to consider a family of models with different parameter values $\text{Θ}={\left[{\text{θ}}_{1}\ldots {\text{θ}}_{N}\right]}^{T}$ compatible with the data *y^m^* , when using the model to predict untested experimental setups. The matrix of parameter values Θ consistent with the data is obtained using *N* realizations of the data obtained by bootstrap (67). Each data realization has the same size as the complete data set, but it is constructed by sampling uniformly from all replicates (3 biological replicates per sampling time). Within each iteration, each replicate has an approximate chance of 37% of being left out, while others might appear several times. The family of solutions, Θ , is then used to make *N* predictions (dynamic simulations) about a given experimental scenario. The median of the simulated trajectories regards the model prediction, while the distribution of the individual solutions at a given sampling time provides a measure of the uncertainty of the model.

### Analysis of dynamic metabolic fluxes.

We selected the most relevant metabolic pathways using a flux ratio, which provides a measure of the net flux over time during growth and stationary phases. In particular, we computed the integral of each flux multiplied by the biomass (millimoles per hour) over time and normalized its value with the accumulated flux of consumed hexoses (glucose and fructose):

(21) $$S_{i,G}=100\times \frac{\int_{t_{L}}^{t_{S}}v_{i}\left(t\right)\cdot \text{DW}\left(t\right)}{\int_{t_{L}}^{t_{S}}v_{\text{Glx}}\left(t\right)\cdot \text{DW}\left(t\right)\;+\;\int_{t_{L}}^{t_{S}}v_{F\text{r}}\left(t\right)\cdot \text{DW}\left(t\right)}$$

(22) $$S_{i,S}=100\times \frac{\int_{t_{S}}^{t_{D}}v_{i}\left(t\right)\cdot \text{DW}\left(t\right)}{\int_{t_{S}}^{t_{D}}v_{\text{Glx}}\left(t\right)\cdot \text{DW}\left(t\right)\;+\;\int_{t_{S}}^{t_{D}}v_{\mathrm{Fr}}\left(t\right)\cdot \text{DW}\left(t\right)}$$

(23) $$S_{i,O}=100\times \frac{\int_{t_{0}}^{t_{F}}v_{i}\left(t\right)\cdot \text{DW}\left(t\right)}{\int_{t_{0}}^{t_{F}}v_{\text{Glx}}\left(t\right)\cdot \text{DW}\left(t\right)\;+\;\int_{t_{0}}^{t_{F}}v_{\mathrm{Fr}}\left(t\right)\cdot \text{DW}\left(t\right)}$$

where *S_i,G_* corresponds to the score of the flux *i* during growth, *S_i,S_* corresponds to the score during the stationary phase, *S_i,D_* corresponds to the score during the decay phase, $v_{i}\left(t\right)$ (millimoles per hour per gram [dry weight]) is the flux under scrutiny, $v_{\mathrm{Glx}}\left(t\right)$ (millimoles per hour per gram [dry weight]) is the flux of glucose, $v_{\mathrm{Fr}}\left(t\right)$ (millimoles per hour per gram [dry weight]) is the flux of fructose, and DW is the predicted dry weight biomass (grams). Results correspond to millimoles of produced compound per millimole of consumed hexose × 100 (mmolH). Score values indicate the overall impact of each reaction in the net oxidation or reduction of electron carriers during the given phase of the fermentation.

### Numerical tools.

To automate the modeling pipeline, we used the AMIGO2 toolbox (68). To solve the dynamic flux balance analysis (dFBA) problem, we used a variable-step, variable-order Adams-Bashforth-Moulton method to solve the system of ordinary differential equations that describe the dynamics of the extracellular metabolites. At each time step, the pFBA problem was solved using the COBRA Toolbox (69). The global optimizer *Enhanced Scatter Search* (eSS [70]) was used to find the optimal parameter values in reasonable computational time.

The ensemble model generation procedure is computationally intensive. However, since each parameter estimation instance in the ensemble is an entirely independent task, we were able to solve this problem in less than a day using 60 CPU cores on a Linux cluster. These tasks were automated with the help of bash scripts and the Open Grid Scheduler. All the scripts necessary to reproduce the results are distributed (https://sites.google.com/site/amigo2toolbox/examples).
