# Supplementary material for: A Multiphase Multiobjective Dynamic Genome-Scale Model Shows Different Redox Balancing among Yeast Species of the Saccharomyces Genus in Fermentation
Source: mSystems. 2021 Aug 3;6(4):e00260-21. doi: 10.1128/mSystems.00260-21 (PMC8407324; doi:10.1128/mSystems.00260-21)
Supplement: TEXT S1 [file msystems.00260-21-t0001.pdf]

SUPPLEMENTAL TEXT 1: Orthology analysis and genome-scale metabolic reconstruction.

Genomes of ScT73, SuBMV58 and SuCECT12600 were sequenced and assembled in previous works [2], Macías et al., (unpublished)). Genome assemblies were annotated by homology and gene synteny using [3]. This approach let us transfer the systematic gene names of *S. cerevisiae* S288c annotation [1] to our assemblies and therefore, to select only those syntenic orthologous genes in T73, CECT12600 and BMV58 genomes for subsequent analyses.

We added to the consensus genome-scale reconstruction of *Saccharomyces cerevisae* S288C (v.8.3.1) metabolites and reactions related to amino acid degradation and higher-alcohols and esters formation. This refined model was then used as a template for reconstructing strain-specific genome-scale models for SuBMV58, SuCECT12600 and ScT73. First, AuReMe was used to generate draft genome-scale metabolic models for each strain using the refined model as a template. As a result, we obtained draft networks that included both gene-associated reactions (supported by genomic evidence and orthology) and non-gene associated reactions, such as transport reactions based on diffusion and exchange reactions, which were assumed to also occur in the strain-specific models. In addition, Metadraft was also used to generate draft networks for each strain using the refined yeast8 as a template. Reactions from MetaDraft were added to the drafts generated with AuReMe. Finally, the models were gap-filled using the refined template of yeast8 as the universe dataset from which reactions are taken to gap-fill the draft models. Figure S1 presents the differences between model reconstructions as compared to the consensus Yeast8.

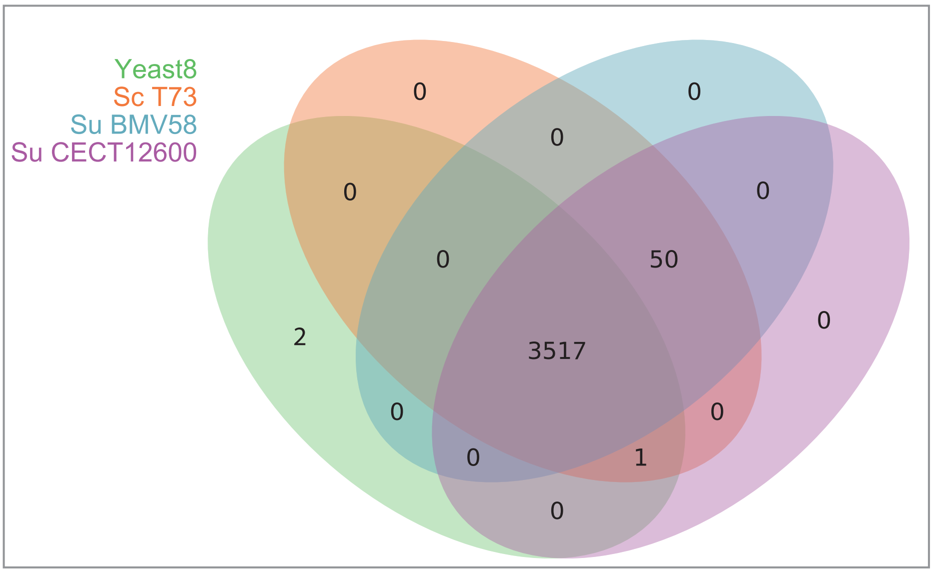

Figure S1: **Differences between model reconstructions as compared to Yeast8.**

References

[1] A Goffeau, B G Barrell, H Bussey, R W Davis, B Dujon, H Feldmann, F Galibert, J D Hoheisel, C Jacq, M Johnston, E J Louis, H W Mewes, Y Murakami, P Philippsen, H Tettelin, and S G Oliver. Life with 6000 genes. *Science*, 274(5287):563–567, 1996.

[2] M. Morard, L. G. Macías, A. C. Adam, M. Lairón-Peris, R. Pérez-Torrado, C. Toft, and E. Barrio. Aneuploidy and ethanol tolerance in *Saccharomyces cerevisiae*. *Front Genet*, 10:82, 2019.

[3] Thomas D Otto, Gary P Dillon, Wim S Degrave, and Matthew Berriman. Ratt: rapid annotation transfer tool. *Nucleic acids research*, 39(9):e57–e57, 2011.
