# Supplementary material for: A Multiphase Multiobjective Dynamic Genome-Scale Model Shows Different Redox Balancing among Yeast Species of the Saccharomyces Genus in Fermentation
Source: mSystems. 2021 Aug 3;6(4):e00260-21. doi: 10.1128/mSystems.00260-21 (PMC8407324; doi:10.1128/mSystems.00260-21)
Supplement: TEXT S2 [file msystems.00260-21-t0002.pdf]

SUPPLEMENTAL TEXT 2: Bootstrap parameter estimation and goodness-of-fit of the model

The model depends on 66 unknown parameters, estimated from the experimental data using a bootstrap approach. The Figure S1 presents the parameter estimation convergence curves for the different strains and bootstrap realizations. Convergence to the optimal solution is in general achieved after 6000 evaluations of the model. The highest distribution of optimal values is observed in SuBMV58 which is induced by a the higher experimental error also found for the experimental data.

Figure S2 presents the bootstrap parameter distributions showing that uncertainty on the parameter estimates was reasonably low. Its value varied between parameters and species. The mean standard deviation corresponds to a 9.5% for the parameter values estimated for ScT73 (excluding the parameter $NGAM$, whose optimal value was zero). A slightly higher mean standard deviation (12.6%) was obtained for the parameter values estimated for SuBMV58 (excluding the kinetic constant describing benzyl alcohol production whose optimal value was zero), and a significantly lower mean uncertainty (2.5%) was obtained for those parameters corresponding to SuCECT12600.

Some parameter values vary significantly between strains (Supplemental Table 3). The comparison between the wine strains ScT73 and SuBMV58, reveals substantial differences - above 100% relative difference, in the growth-associated ATP maintenance; the rate of transport of specific amino acids and hexoses and the production of certain alcohols and acids. GAM_{fitted} for SuBMV58 is around twice the value for ScT73. The rate of transport of phenylalanine, leucine, alanine and cysteine is between 106% and 778% larger for SuBMV58 than for ScT73. Similarly, hexoses uptake rate is around two times higher at the beginning of fermentation for SuBMV58 than for ScT73.

Regarding products, the most noticeable difference appears in the production rate of succinate and, to a lesser extent, in the production of 2-phenylethanol, 2-phenylethyl acetate, and 2,3-butanediol. The comparison between the wine strains and the natural strain showed that the lag phase is more than three times longer for the natural strain; besides, the non-growth associated maintenance is practically zero for wine strains while it is around 0.8 for the SuCECT12600 strain. The rates of hexoses uptake are pretty similar between *S. uvarum* strains; on the contrary, we found substantial differences in the uptake of various amino acids. In particular, the uptake rate for threonine is 740% higher for SuCECT12600 than for SuBMV58.

The Figure S3 shows the best fit to the data. We determined the R-squared measure of goodness of fit (R^2) for each measured variable and each strain-based fermentation. The Supplemental Table 3 presents the corresponding values. The vast majority of the coefficients were positive with few

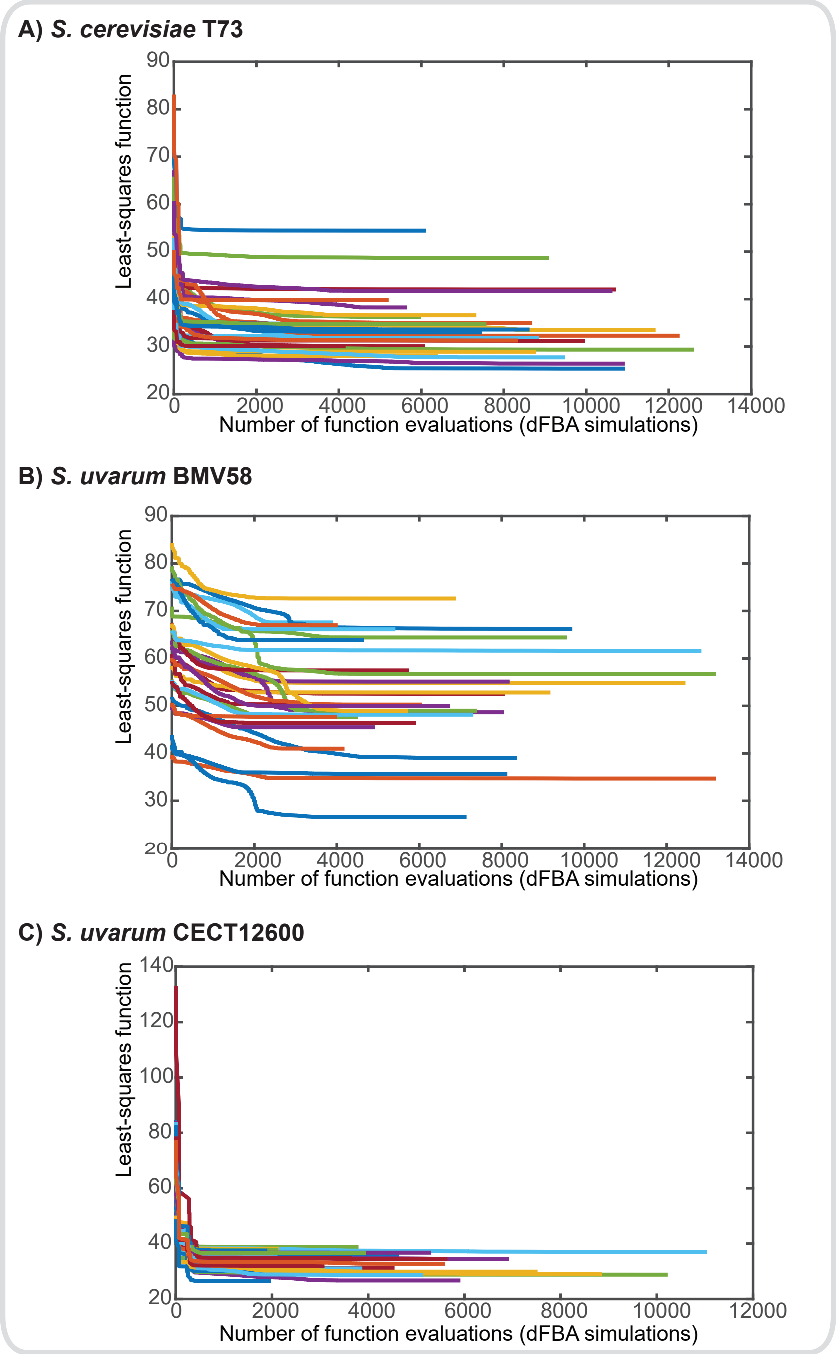

Figure S1: **Convergence of the bootstrap approach for the strain-specific parameter estimation.**

exceptions (10 out 141), typically associated with low signal-to-noise ratio and high data dispersion observed in the measured variable, e.g., cysteine. The mean R^2 (excluding negative values) was of 0.88 for *S. cerevisiae* T73, 0.92 for *S. uvarum* BMV58 and 0.91 for *S. uvarum* CECT12600; the median R^2 values are above 0.94 for all strains.

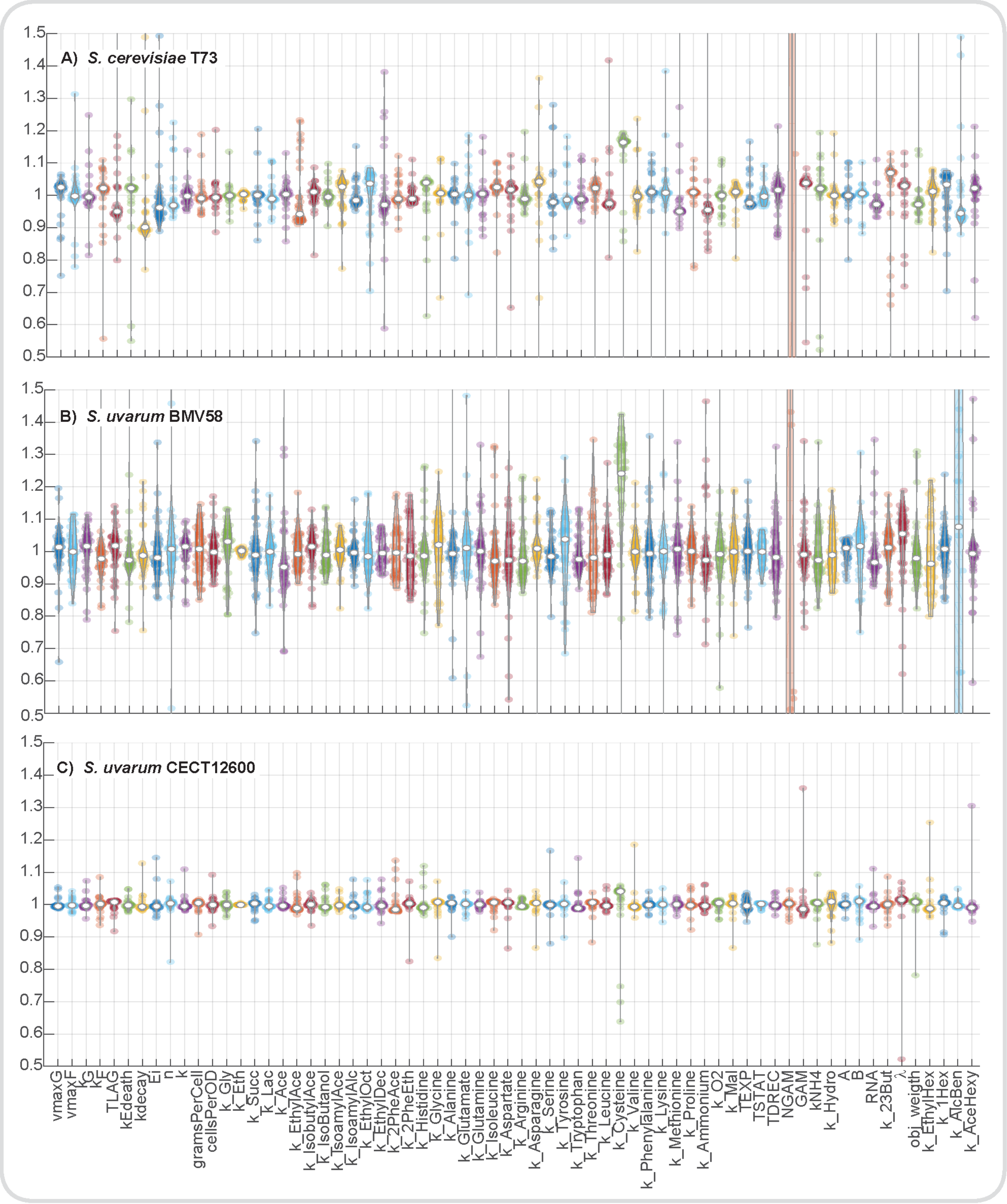

Figure S2: **Distribution of parameter values as obtained by the bootstrap approach. The normalized values are computed by dividing each best solution by the mean of all solutions.**

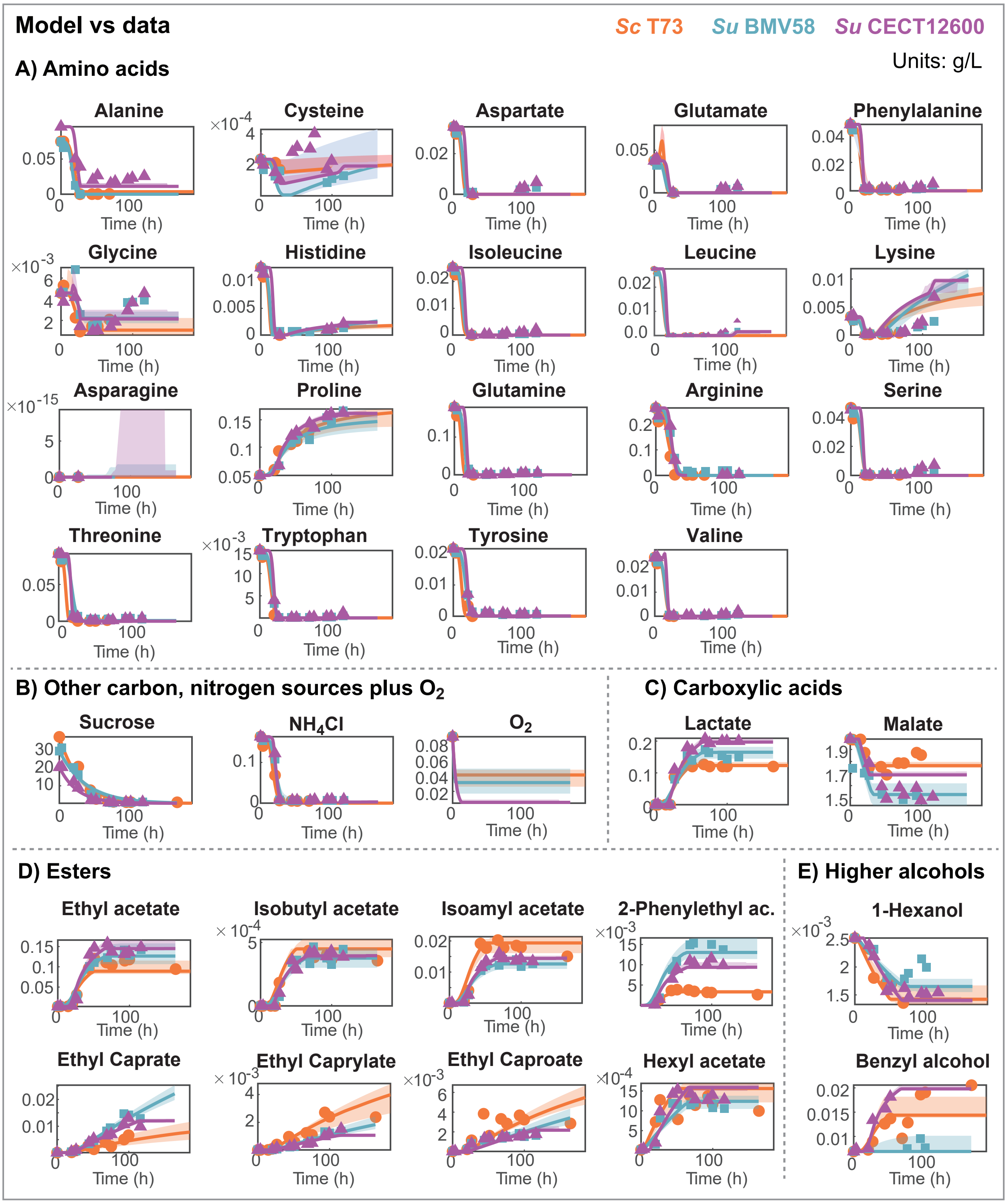

Figure S3: **Best fit to the data and model associated undertainty**. Figures show the data (symbols) and the model (continuous lines) for several measured variables: nutrients and products. Shading areas correspond to model uncertainty as predicted by the bootstrap approach. The quality of model is visually good. Besides goodness of fit as measured by R^2 was obtained for each measured quantity. The vast majority of the R^2 values were positive with few exceptions (10 out 141), typically associated with low signal-to-noise ratio and high data dispersion observed in the measured variable, e.g., cysteine. The mean R^2 (excluding negative values) was of 0.88 for *S. cerevisiae* T73, 0.92 for *S. uvarum* BMV58 and 0.91 for *S. uvarum* CECT12600.
