# Supplementary material for: A Multiphase Multiobjective Dynamic Genome-Scale Model Shows Different Redox Balancing among Yeast Species of the Saccharomyces Genus in Fermentation
Source: mSystems. 2021 Aug 3;6(4):e00260-21. doi: 10.1128/mSystems.00260-21 (PMC8407324; doi:10.1128/mSystems.00260-21)
Supplement: TEXT S3 [file msystems.00260-21-t0003.pdf]

SUPPLEMENTAL TEXT 3: Detailed description of the redox balance mechanisms used by the different strains.

The comparative analysis of dynamic flux ratios showed that most significant differences between strains occur in the stationary phase. Figure S1 presents the relative differences between the fluxes obtained for *S. uvarum* species and those obtained for *S. cerevisiae*. Only those fluxes resulting in a relative difference above 100% are shown. Remarkably, the figure illustrates that not only a significantly higher number of fluxes differ in the stationary phase but also, the relative difference is also higher.

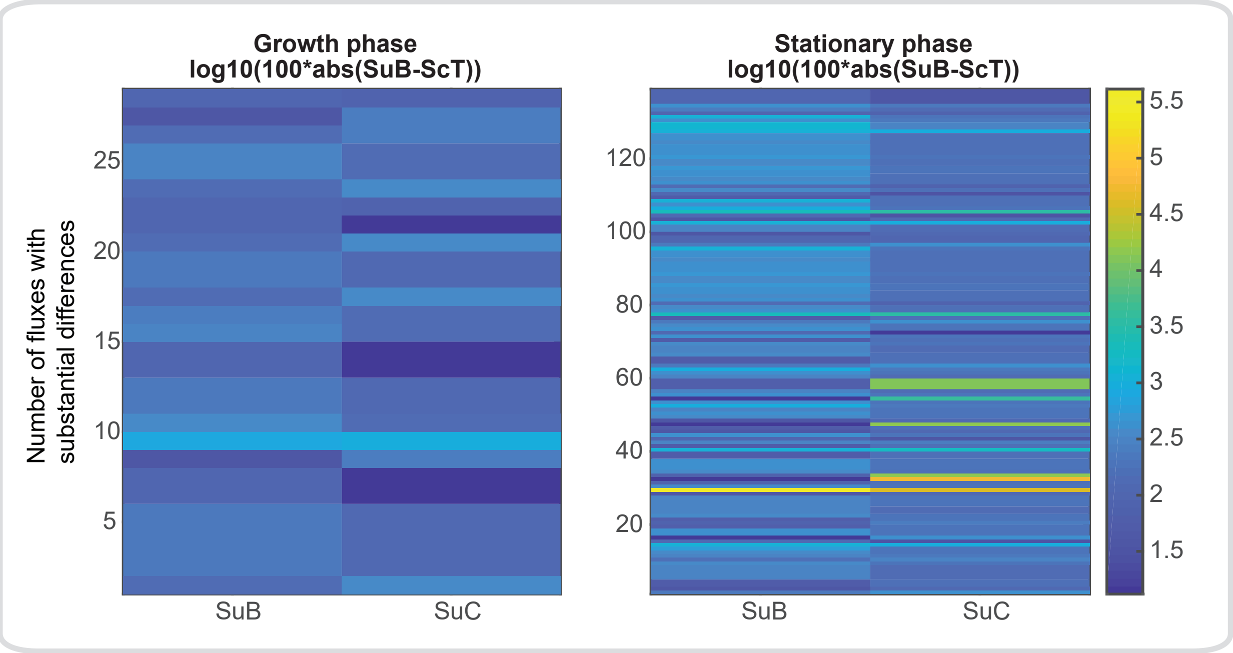

Figure S1: **Comparison of fluxes between growth and stationary phases**. Figure shows relative differences of *S. uvarum* species with respect to *S. cerevisiae*. Only those relative differences above 100% are shown. Note that while for the growth phase only 29 fluxes are between $100 - 150\%$ relative difference; for the stationary phase more than 140 fluxes differ between the $100 - 550\%$.

Our results show that *S. uvarum* and *S. cerevisiae* strains use different redox balance strategies markedly visible during the fermentation stationary phase. Here we present a detailed view of the differences found in the central carbon metabolism and the production of higher alcohols.

Central carbon metabolism

Figure S2 presents the dynamic flux ratios during the stationary phase of the most relevant pathways in the central carbon metabolism, including glycolysis and their impact on redox couple co-factors NADH/NAD+ and NADPH/NADP+.

Production of ethanol. Taking the entire course of the fermentation in consideration, *S. uvarum* strains had a lower ethanol production rate than *S. cerevisiae* strain (187.40, 179.14, 182.40 *mmol*/*mmolH* in ScT73, SuBMV58 and SuCECT12600, respectively; Table S4, r_2115). Also, the model predicted slightly lower ethanol rates during growth phase for the three strains (173.10, 177.24, 183.34 *mmol*/*mmolH*), while they were quite similar during stationary phase (186.86, 177.24, 183.34 *mmol*/*mmolH*) (Figure S2). Part of the 'missing' carbon was to be found in the yield of glycerol and other downstream pathways using pyruvate as substrate.

Production of glycerol. During the growth phase, the two *S. uvarum* strains had higher glycerol production rates, with SuBMV58 and SuCECT12600 strains producing 8.37 and 8.34 *mmol*/*mmolH* respectively, while the ScT73 strain only produced 7.25 *mmol*/*mmolH* (Table S4, r_0489). Afterward, the model predicted a smaller but still significant difference in glycerol production during the stationary phase (7.09, 7.84 and 7.81 *mmol*/*mmolH* for ScT73, SuBMV58 and SuCECT12600 respectively; Figure S2). Consistent with this, the overall score for the production of glycerol was lower for ScT73 (4.21 *mmol*/*mmolH*) than for both *S. uvarum* ($> 6 mmol/mmolH$) (Table S4, r_0489). As an NADH-consuming process, biosynthesis of glycerol plays an essential role in maintaining cytosolic redox balance during anaerobic conditions by oxidizing excess NADH to NAD+. Glycerol is also the only compatible solute to counterbalance the osmotic pressure in *S. cerevisiae* with glucose as a carbon source [1]. Directed evolution experiments exposing *S. cerevisiae* to osmotic stress led strains to produce a higher amount of glycerol, 2,3-butanediol, and succinate [5]. In winemaking conditions, cells suffer hyperosmotic stress due to the elevated amount of sugars. The fact that *S. uvarum* strains present a higher amount of extracellular glycerol may indicate that *S. cerevisiae* tends to accumulate intracellular glycerol at early times during fermentation as previously reported by Perez-Torrado et al. [4].

Production of succinate. The model predicted that the fraction of pyruvate incorporated into the mitochondria during the stationary phase accounted for 3.22, 3.64, 2.34 *mmol*/*mmolH* in ScT73, SuBMV58 and SuCECT12600 respectively (r_2034). Once inside mitochondria, pyruvate was either used for acetyl-CoA formation (1.37, 2.49, 1.43,*mmol*/*mmolH* for ScT73, SuBMV58 and SuCECT12600 respectively, r_0961) or directed towards the *de novo* production of valine and isobutanol (0.95, 0.59, 0.50, *mmol*/*mmolH* Figure).

Consistent with succinate raw data, the model estimated a significantly higher production rate of this by-product on the overall fermentative process in SuBMV58 (3.59 *mmol*/*mmolH*) and SuCET12600 (1.10 *mmol*/*mmolH*) than ScT73 (0.35 *mmol*/*mmolH*) (Table S4, r_2057). Remarkably, the difference in production between *S. uvarum* strains was noteworthy. We also reported

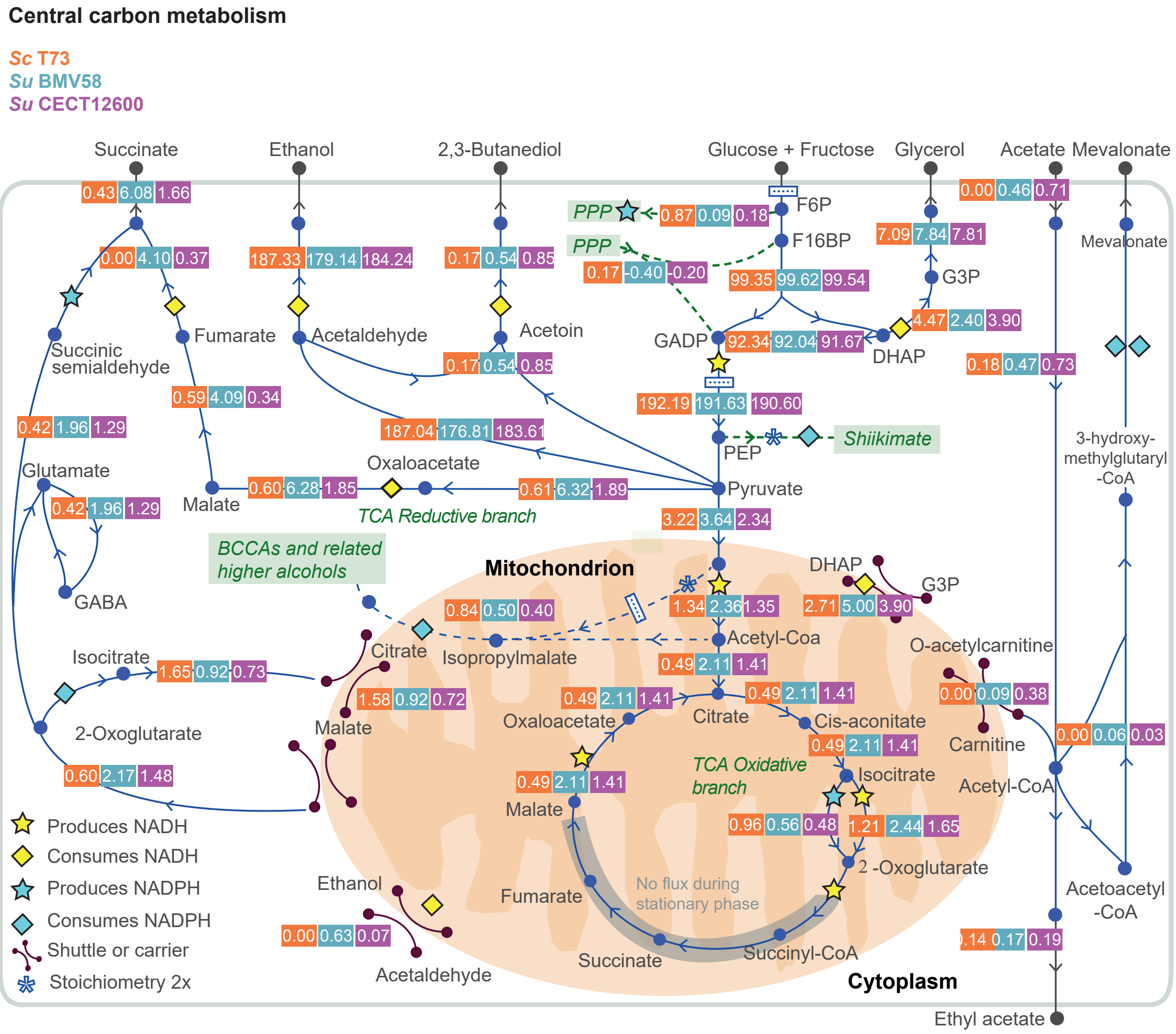

Figure S2: **Redox balance in Central Carbon Metabolism**. Figure shows the predicted intracellular dynamic flux ratios associated with the central carbon metabolism during the stationary phase.

interesting variations in the rate and the origin of succinate according to the fermentation phase between strains. In anaerobic conditions during growth and stationary phases, there are three possible routes of producing succinate: the oxidative and reductive branch of the tricarboxylic acid cycle (TCA) and the GABA shunt. In anaerobic conditions, the TCA is truncated at the succinate level: TCA does not work cyclically but follows either a mitochondrial oxidative branch or a cytoplasmic reductive branch (Figure S2). In the former case, succinate is produced from pyruvate via four cytoplasmic reactions, yielding two NAD+ per pyruvate. In the other case, pyruvate is internalized into mitochondria and oxidized until succinate, producing three NADH per pyruvate. As for the GABA shunt, it is the pathway that circumvents the formation of succinyl-CoA in the oxidative branch of the TCA cycle. Instead, it converts 2-oxoglutarate into succinate with the intermediaries glutamate, GABA, and succinate semi-aldehyde, yielding two NADH and one NADPH per pyruvate (Figure S2). In this regard, our model predicted that during the growth phase, a carbon flux existed through the oxidative branch of TCA, but only until 2-oxoglutarate in the three strains and that there was no flux through the GABA shunt (Table S4). Therefore, the succinate formed during growth was yielded only by the reductive branch of TCA in ScT73, SuBMV58 and SuCECT12600. However, the most significant fraction of succinate was produced during the stationary phase, and with a significant higher rate by SuBMV58 (6.08 $mmol/mmolH$) and SuCECT1600 (1.66 $mmol/mmolH$) than ScT73 (0.43 $mmol/mmolH$). Interestingly, the model predicted that during the stationary phase, the GABA pathway (0.42, 1.96 and 1.29 $mmol/mmolH$ for ScT73, SuBMV58 and SuCECT12600 respectively; Figure **??**) summed to the reductive branch of TCA (0.00, 4.10 and 0.37 $mmol/mmolH$ for ScT73, SuBMV58 and SuCECT12600 respectively; Figure S2). Once again, the model suggested that during the stationary phase, the TCA oxidative branch was active until 2-oxoglutarate and the GABA shunt was responsible for completing the conversion of pyruvate-derived acetyl-CoA into succinate. Remarkably, the contribution of the TCA reductive branch succinate formation was almost twice that of the GABA shunt in SuBMV58, while it was almost equal in ScT73 and SuCECT12600 strains. Also, as shown in Figure S2, the model suggested that the NADH produced in the oxidative branch of the TCA cycle could be re-oxidized at the level of the mitochondrial shuttles responsible for: i) the reduction of cytoplasmic DHAP to G3P (2.71, 5.00 and 3.90 $mmol/mmolH$), ii) the reduction of cytoplasmic acetaldehyde to ethanol (0, 0.63 and 0.07 $mmol/mmolH$).

The finding that the GABA shunt would contribute to succinate formation in the case of *S. uvarum* strains was somewhat unexpected given previous results on *S. cerevisiae.* bach2009role concluded that for *S. cerevisiae* the GABA shunt was of little relevance on redox metabolism and that glutamate decarboxylase (GAD1) was poorly expressed when wine succinate is produced. Nevertheless, [3] observed substantial differences regarding intracellular levels of GABA in cryotolerant

species. This fact and our results indicate that the production of succinate among *Saccharomyces* species requires further investigation.

Production of 2,3-butanediol. *S. uvarum* strains were also more active in the production of 2,3-butanediol -a fermentative by-product involved in NADH oxidation from acetoin- than *S. cerevisiae*. The model correctly fitted extracellular raw data and consistently predicted higher flux toward 2,3-butanediol production in *S. uvarum* strains during growth (0.22, 0.55, 0.98 $mmol/mmolH$ in ScT73, SuBMV58 and SuCECT12600, respectively, r_1097) and stationary phases (0.17, 0.54 and 0.85 $mmol/mmolH$ in ScT73, SuBMV58 and SuCECT12600, respectively). Again, but inversely to succinate, the flux difference towards 2,3-butanediol production between *S. uvarum* strains was noteworthy (Figure S2). Because both succinate and 2,3-butanediol generate from pyruvate, our results indicate that the two *S. uvarum* strains might use two different carbon redirection strategies around the pyruvate node: SuCECT12600 directing a larger fraction of pyruvate to the synthesis of 2,3-butanediol, and SuBMV58 to the synthesis of succinate.

Production and consumption of acetate. Finally, we observed another striking difference between the three strains in the dynamics of acetate. As shown in Figure 3 in the main text, the three strains produced acetate during the growth phase and until the entry into the stationary phase. Afterward, while extracellular acetate concentration remained constant in ScT73, it decreased in both *S. uvarum* strains indicating an acetate consumption. Our model successfully described these phenotypes. On the first hand, we reported quite similar flux towards acetate production during the growth phase in the three strains (1.08, 1.14 and 1.42 $mmol/mmolH$ in ScT73, SuBMV58 and SuCECT12600, respectively; Table S4, r_1106). On the other hand, during the stationary phase, we obtained that SuBMV58 and SuCECT12600 strains consumed acetate with a rate of -0.46 and -0.71 $mmol/mmolH$ respectively; on the contrary, ScT73 displayed an acetate rate equal to 0 $mmol/mmolH$ (Table S4, r_1106). Considering the entire fermentation process, we determined an overall acetate consumption of -0.12 $mmol/mmolH$ for SuCECT12600, while a limited production of 0.036 $mmol/mmolH$ was computed for SuBMV58. In the case of the ScT73 strain, this production was five times higher (0.17 $mmol/mmolH$; Table S4,r_1106).

In our first simulations, the model determined that *S. uvarum* strains used a significant part of this consumed acetate to produce succinate through the glyoxylate pathway. This result made sense from a pFBA point of view because this path resulted in the smallest overall flux throughout the metabolic network. However, we could not find literature supporting the production of glyoxylate in the presence of a large glucose concentration. Therefore, we decided to constrain the isocitrate lyase flux to zero. The revised model then suggested that during stationary phase SuBMV58 and

SuCECT12600 strains incorporated the acetate derivative, acetyl-CoA, into ethyl acetate in the cytoplasm (0.17 and 0.19 $mmol/mmolH$); shifted most of the remaining fraction (0.09 and 0.38 $mmol/mmolH$) into the mitochondria through the carnitine shuttle system and the last part (0.06 and 0.03) was directed towards mevalonate (Figure S2). The fact that an amount of the acetate carbon was directed towards mevalonate is in line with recent experimental work by [?].

Inside the mitochondria, acetyl-CoA was used to form isopropylmalate (precursor of isoamyl alcohol, Figure or further used in the TCA towards the synthesis of 2-oxoglutarate (Figure S2).

Contributions to redox balance. All the aforementioned fermentative by-products (ethanol, glycerol, succinate, 2,3-butanediol and acetate) impact NADH/NAD+ and/or NADPH/NADP+ balance (Figure S2). However, their relative contribution to maintaining co-factors equilibrium varies according to the stoichiometry of the reaction, the function of the cell compartment in which they are produced (cytoplasm or mitochondria) and the activity of redox shuttles between compartments. Most of the glycolytic pyruvate was directed towards ethanol production for the three strains, known to be redox neutral. However, we noticed that both glycerol synthesis and reductive succinate production were more pronounced in *S. uvarum* strains during the stationary phase. Thus, according to the stoichiometry and localization of these pathways, it may result in a cytoplasmic surplus of NAD+ that should be compensated elsewhere in the metabolism of *S. uvarum* strains.

Production of higher alcohols

Higher alcohol production started during the growth phase and ceased at the end of the stationary phase. Our results reflect substantial differences in the accumulation of some higher alcohols between strains. In particular, the model predicted that the carbon skeletons of isoamyl alcohol, isobutanol and 2-phenyl ethanol were in a significant part synthesized *de novo* from glycolytic and pentose phosphate pathway intermediates, rather than coming from the catabolism of precursor exogenous amino acids (leucine, valine and phenylalanine respectively). Furthermore, the model shows that isoamyl alcohol and 2-phenyl ethanol contribute to glycerol formation in wine fermentation.

Figure shows the predicted intracellular fluxes related to higher alcohols during the stationary phase and its corresponding impact on the redox co-factors balance NADPH/NADP+ and NADH/NAD+. Readers can find the dynamic flux ratios in Table S4.

During the stationary phase, *S. uvarum* strains produced more 2-phenylethanol than ScT73 strain per unit of consumed hexoses (0.16, 0.66 and 0.38 $mmol/mmolH$ for ScT73, SuBMV58 and SuCECT12600 respectively, r_1590) while the opposite pattern was observed for isoamyl alcohol

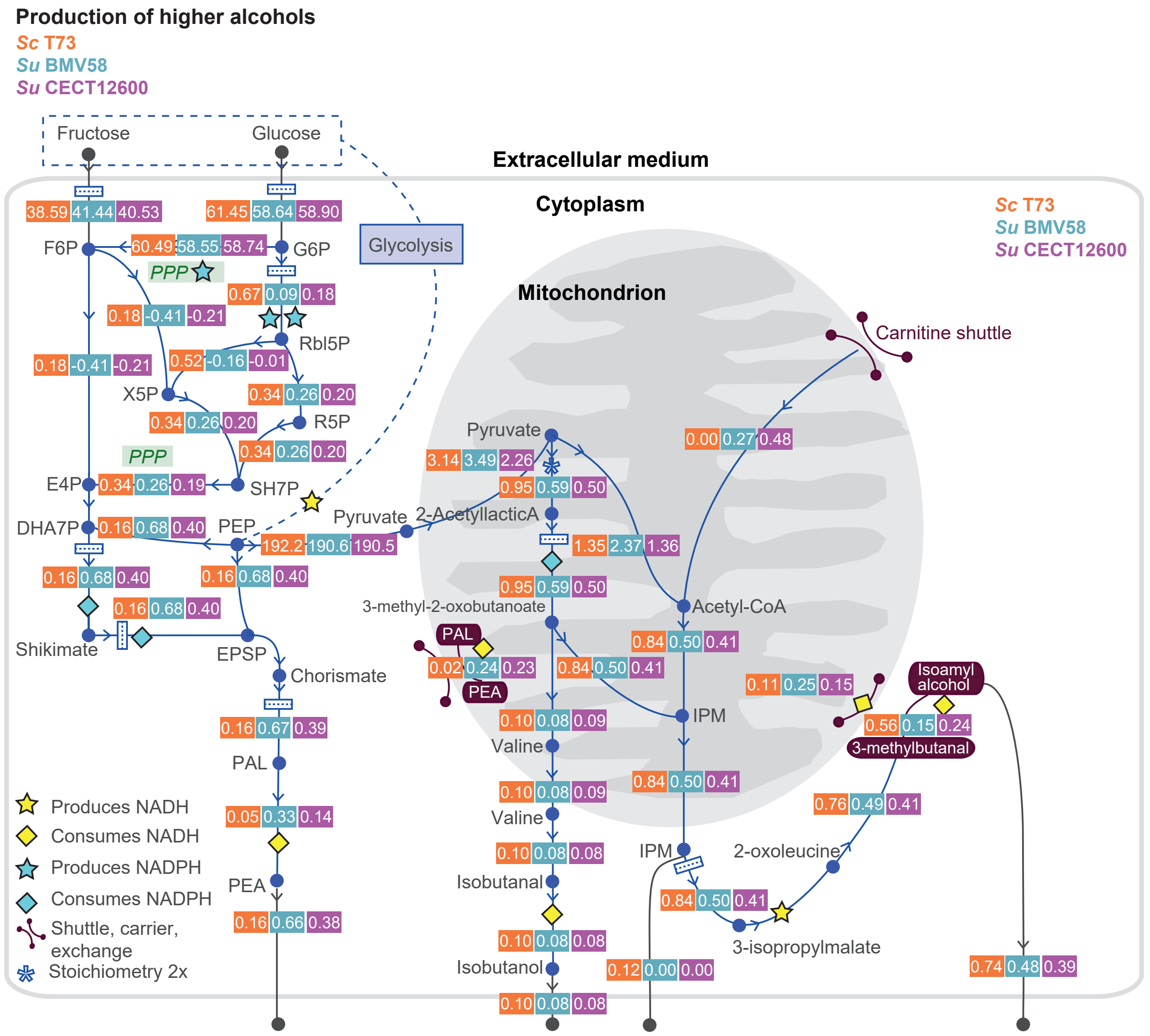

Figure S3: **Redox balance in production of higher alcohols.**. Figure shows the predicted intracellular fluxes related to higher alcohols during the stationary phase and its corresponding impact on the redox co-factors balance NADPH/NADP+ and NADH/NAD+.

(0.74, 0.48 and 0.39 $mmol/mmolH$ for ScT73, SuBMV58 and SuCECT12600 respectively, r_1863). In contrast, the model prediction was quite similar for the three strains for isobutanol ($\simeq$ 0.09 $mmol/mmolH$), as well as for other higher alcohols such as methionol and tyrosol which seemed to accumulate in minimal quantities ($\simeq$ 0.001 $mmol/mmolH$) in response to perturbations in the amino acid pool.

Production of 2-phenylethanol. *De novo* synthesis of 2-phenylethanol (PEA, Figures S3.A, S3.B) from carbohydrates contributed to redox homeostasis. Erythrose-4-phosphate (E4P, Figure) and phosphoenolpyruvate (PEP) are two sugar-phosphate intermediates of the pentose phosphate pathway (PPP) and glycolysis. They are the starting substrates of the chorismate pathway that lead to phenylalanine, which can subsequently be catabolized to 2-phenylethanol (Ehrlich pathway). From the beginning of the chorismate pathway to 2-phenylethanol, one NADPH (3-dihydroshikimate $\rightarrow$ shikimate) and one NADH (phenylacetaldehyde (PAL) $\rightarrow$ PEA) are consumed (Figure).

However, to fully understand the impact of *de novo* production of 2-phenylethanol on redox homeostasis, it is also important to remember that the PPP consists of two branches: i) an oxidative and irreversible branch from glucose-6-phosphate (G6P) to ribulose-5-phosphate (Rbl5P) resulting in the net formation of two reduced NADPH co-factors per molecule of glucose-6-phosphate (Figure), and ii) a non-oxidative branch consisting of reversible carbon shuffling reactions between sugar-phosphate molecules leading to important precursor metabolites (e.g., ribose-5-phosphate (R5P) and erythrose-4-phosphate (E4P)) and glycolytic intermediates (e.g., fructose-6-phosphate (F6P) and glyceraldehyde-3-phosphate (G3P)). Thus, the transketolase and transaldolase enzymes of this branch of the PPP provide a reversible link between the PPP and glycolysis [2].

In this context, if E4P required for *de novo* synthesis of PEA was generated through the oxidative branch of PPP, PEA production would result in NADPH accumulation. However, if E4P was generated by the non-oxidative branch of PPP from sugar phosphate intermediates of the glycolysis, we would expect an NADP+ accumulation.

As shown in Figure , our model predicted that during stationary phase ScT73 had a greater flux through the oxidative branch of the PPP than *S. uvarum* strains (0.67, 0.09 and 0.18 $mmol/mmolH$ for ScT73, SuBMV58 and SuCECT12600 respectively). On the contrary, dynamic flux ratios through several non-oxidative reactions of the PPP were higher in both *S. uvarum* strains (F6P $\rightarrow$ E4P : 0.18, -0.41 and -0.21 $mmol/mmolH$; F6P $\rightarrow$ X5P :0.18, -0.41 and -0.21 $mmol/mmolH$; Figure). Consistent with an higher 2-phenylethanol synthesis by SuBMV58 and SuCECT12600, the model predicted an increase in flux towards chorismate synthesis in both *S. uvarum* strains (0.17, 0.68 and 0.40 $mmol/mmolH$ for ScT73, SuBMV58 and SuCECT12600, respectively). The higher

flux through the PPP oxidative branch in ScT73 can be partially explained by NADPH requirement (at the level of 3-methyl-2-oxobutanoate formation) in *de novo* synthesis of isoamyl alcohol. On the other hand, the alternative non-oxidative PPP strategy used by *S. uvarum* strains may contribute to providing $NADP^+$ co-factors required in the $NADP^+$-dependent glutamate degradation to succinate in the GABA shunt (0.42, 1.96 and 1.29 $mmol/mmolH$ for ScT73, SuBMV58 and SuCECT12600, respectively; Figure).

Production of isoamyl alcohol. During the stationary phase, the model predicted a substantial flux from pyruvate to 2-acetyllactic acid inside the mitochondria (0.95, 0.59 and 0.50 $mmol/mmolH$ for ScT73, SuBMV58 and SuCECT12600 respectively; Figure ; r_0097). In this reaction, two pyruvates are required per 2-acetyllactic molecule formed. The model also suggested that 2-acetyllactic acid was mainly directed to forming 3-isopropylmalate (IPM, 0.84, 0.50 and 0.41 mmol/mmolH for ScT73, SuBMV58 and SuCECT12600 respectively; Figure ; r_0025), consuming one NADPH and one Acetyl-CoA. 3-isopropylmalate was mainly converted in isoamyl alcohol (0.76, 0.49 and 0.41 mmol/mmolH for ScT73, SuBMV58 and SuCECT12600 respectively) rather than for *de novo* leucine synthesis. The conversion of 3-isopropylmalate into 2-oxoleucine releases one NADH (r_0061), consumed during the reduction of 3-methylbutanal to isoamyl alcohol (Figure , r_0179). Following these reactions, the formation of each isoamyl alcohol molecule consumes two pyruvate molecules, one acetyl-CoA and one NADPH (Figure).

Besides, the formation of two pyruvates from one glucose release two NADH, and two NADH are produced per acetyl-CoA formed. Summing up, *de novo* synthesis of one isoamyl alcohol should result in excess of four NADH and one $NADP^+$. Accordingly, this *de novo* synthesis of isoamyl alcohol from pyruvate has a relevant impact on redox balance. The citrate/2-oxoglutarate could provide the $NADP^+$ required to synthesize 3-methyl-2-oxobutanoate inside the mitochondrion NADPH shuttle.

Remarkably, the production of higher alcohols contributed substantially to redox metabolism related to glycerol accumulation. During the stationary phase, approximately 43% of the glycerol produced by the ScT73 strain was attributable to NADH derived from isoamyl-alcohol and 2-phenyl ethanol production. In the cases of SuBMV58 and SuCECT12600 strains, these values dropped to 36% and 27%, respectively.

References

[1] R. Babazadeh, P.-J. Lahtvee, C. Adiels, M. Goksor, J. B. Nielsen, and S. Hohmann. The yeast osmostress response is carbon source dependent. *Sci Rep*, 7:990, 2017.

[2] Jeremy M Berg. Biochemistry 5th edition, 2006.

[3] M. López-Malo, A. Querol, and J. M. Guillamon. Metabolomic comparison of *Saccharomyces cerevisiae* and the cryotolerant species *S bayanus var. uvarum* and *S. kudriavzevii* during wine fermentation at low temperature. *PloS One*, 8(3):e60135, 2013.

[4] R. Pérez-Torrado, B. M. Oliveira, J. Zemančíková, H. Sychrová, and A. Querol. Alternative glycerol balance strategies among *Saccharomyces species* in response to winemaking stress. *Frontiers in Microbiol.*, 7:435, 2016.

[5] V. Tilloy, A. Ortiz-Julien, and S. Dequin. Reduction of ethanol yield and improvement of glycerol formation by adaptive evolution of the wine yeast *Saccharomyces cerevisiae* under hyperosmotic conditions. *Appl. Environ. Microbiol.*, 80(8):2623–2632, 2014.
